# Landscape of Targeted Anti-Cancer Drug Synergies in Melanoma Identifies a Novel BRAF-VEGFR/PDGFR Combination Treatment

**DOI:** 10.1371/journal.pone.0140310

**Published:** 2015-10-13

**Authors:** Adam A. Friedman, Arnaud Amzallag, Iulian Pruteanu-Malinici, Subash Baniya, Zachary A. Cooper, Adriano Piris, Leeza Hargreaves, Vivien Igras, Dennie T. Frederick, Donald P. Lawrence, Daniel A. Haber, Keith T. Flaherty, Jennifer A. Wargo, Sridhar Ramaswamy, Cyril H. Benes, David E. Fisher

**Affiliations:** 1 Massachusetts General Hospital Cancer Center, Harvard Medical School, Boston, Massachusetts, United States of America; 2 Dermatology and Cutaneous Biology Research Center, Massachusetts General Hospital, Charlestown, Massachusetts, United States of America; 3 Department of Surgical Oncology, University of Texas M.D. Anderson Cancer Center, Houston, Texas, United States of America; 4 Department of Genomic Medicine, University of Texas M.D. Anderson Cancer Center, Houston, Texas, United States of America; 5 Division of Dermatopathology, Massachusetts General Hospital, Boston, Massachusetts, United States of America; 6 Howard Hughes Medical Institute, Chevy Chase, Maryland, United States of America; Rutgers University, UNITED STATES

## Abstract

A newer generation of anti-cancer drugs targeting underlying somatic genetic driver events have resulted in high single-agent or single-pathway response rates in selected patients, but few patients achieve complete responses and a sizeable fraction of patients relapse within a year. Thus, there is a pressing need for identification of combinations of targeted agents which induce more complete responses and prevent disease progression. We describe the results of a combination screen of an unprecedented scale in mammalian cells performed using a collection of targeted, clinically tractable agents across a large panel of melanoma cell lines. We find that even the most synergistic drug pairs are effective only in a discrete number of cell lines, underlying a strong context dependency for synergy, with strong, widespread synergies often corresponding to non-specific or off-target drug effects such as multidrug resistance protein 1 (MDR1) transporter inhibition. We identified drugs sensitizing cell lines that are BRAF^V600E^ mutant but intrinsically resistant to BRAF inhibitor PLX4720, including the vascular endothelial growth factor receptor/kinase insert domain receptor (VEGFR/KDR) and platelet derived growth factor receptor (PDGFR) family inhibitor cediranib. The combination of cediranib and PLX4720 induced apoptosis *in vitro* and tumor regression in animal models. This synergistic interaction is likely due to engagement of multiple receptor tyrosine kinases (RTKs), demonstrating the potential of drug- rather than gene-specific combination discovery approaches. Patients with elevated biopsy KDR expression showed decreased progression free survival in trials of mitogen-activated protein kinase (MAPK) kinase pathway inhibitors. Thus, high-throughput unbiased screening of targeted drug combinations, with appropriate library selection and mechanistic follow-up, can yield clinically-actionable drug combinations.

## Introduction

Although response rates within genetically-selected subpopulations of solid tumor cancer patients can be high, such as 60–80% among *BRAF* ^*V600E*^ mutant melanoma patients receiving the BRAF inhibitor vemurafenib [1], few patients achieve single-agent complete responses. Thus, a significant number of patients have intrinsic resistance to MAPK pathway inhibition. Even among patients who do respond, most will develop acquired resistance within a year, often due to additional mutations or bypass pathways [2, 3]. Recently several groups have discovered mechanisms of acquired resistance to BRAF-targeted therapy, usually in initially sensitive cell lines such as A375 [4–7], pointing to the complexity of identifying salvage therapeutic strategy and few studies have addressed *de novo* resistance to vemurafenib in the context BRAF^V600E^ [8]. Drug combinations have the potential to address *de novo* and acquired resistance but predicting drug combination activity from single agents is not yet feasible in part because only relatively small datasets of combination exist. Candidate-based discovery of combination drug targets such as sequencing tumors for additional driver somatic mutations [8] or unbiased RNAi or cDNA screens can yield actionable targets. However, these approaches may miss potential high-order interactions with inhibitors targeting multiple proteins and their clinical relevance may depend upon lengthy drug discovery efforts around novel targets. Moreover, based on strong context dependency seen for single agent activity it is expected that combinations’ activity and synergism will also be context specific. However, it is not yet clear whether combinations of targeted agents could be efficacious across a broad range of tumor subtype, making them applicable to more patients than their single agent constituent or whether resistance needs to be addressed by a large number of context specific combinations addressing smaller groups of patients than the constituting single agents. Several groups have started to identify drug-drug interactions in an unbiased way in cancer cells [9, 10], which have yielded important insights. We have previously described massively-scaled single-agent drug screening across a large panel of genotypically-defined cancer cell lines [11]. To understand the overall landscape and potential of scaled drug-drug interaction screening across cancer cell lines as an initial phase of a Cancer Cell-line Combination (C^3^) project, we screened a large collection of melanoma cell lines across several thousand combinations of targeted inhibitors. Melanoma was selected in light of the availability of a large number of cell lines harboring a common mutated oncogene (BRAF^V600E^) and a validated targeted therapy.

## Results

### Systematic combination drug synergy discovery

**Fig 1:**
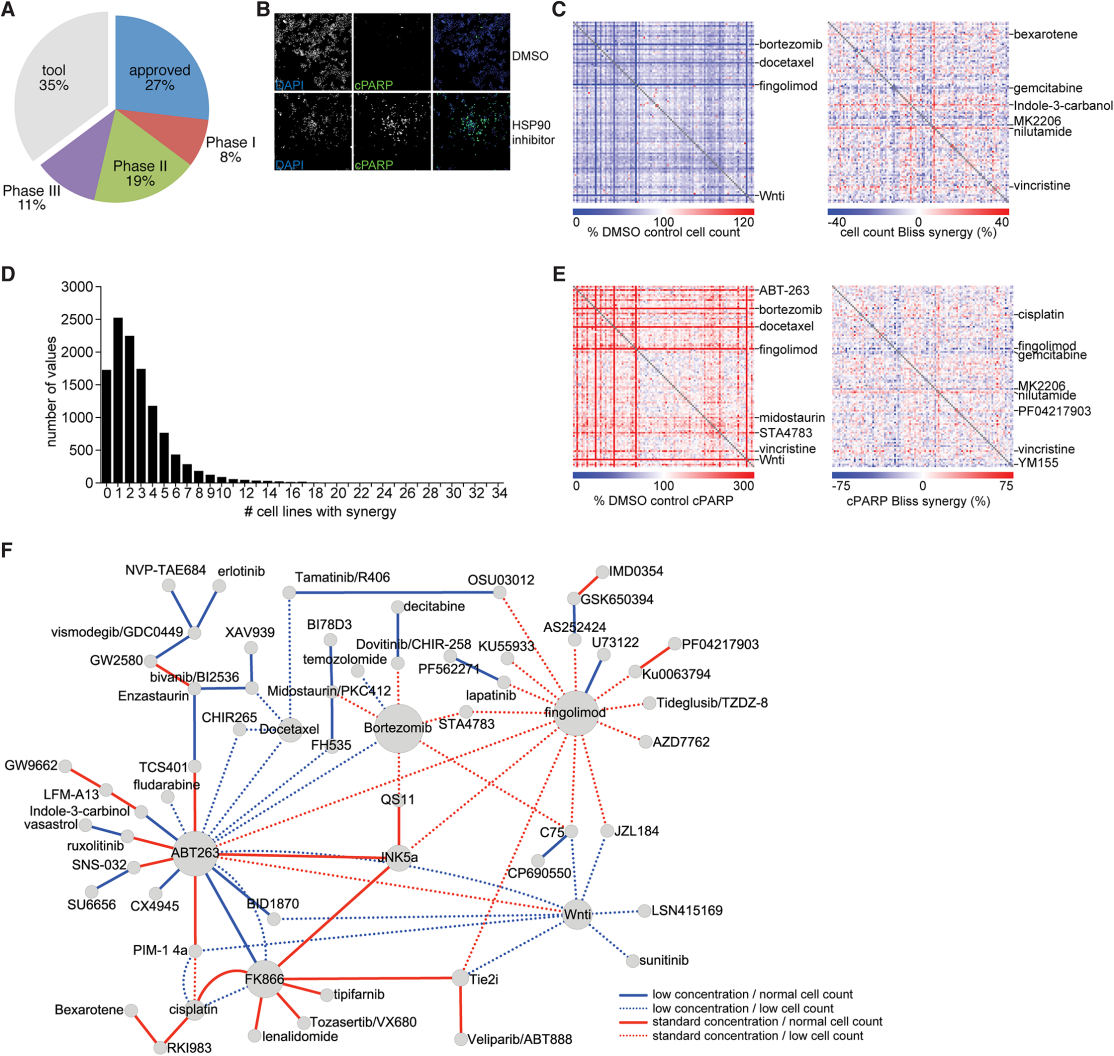
Ultra high-throughput screen to identify synergistic combinations in melanoma cells. (A) Summary of clinical development stage of 108 drugs included in the combination drug panel. (B) Example of raw UHTS data generated, demonstrating cell count information collected from DAPI channel and apoptosis data from cleaved PARP immunofluorescence; positive control of HSP90 inhibitor 17-AAG treatment is shown. (C) Summary matrix of combinatorial drug data, with each point representing the effect of one of 5,778 combinations at the standard drug concentration, as the median effect of the drug combination across all 36 melanoma cell lines on the relative cell count (left) and the calculated Bliss synergy score for that combination (right). (D) Histogram of number of cell lines a given drug combination showed synergy. Peak number of synergies were seen in one cell line, indicating many synergies are private. (E) As in (C), showing median effect of the drug combination (at standard concentration) on the relative cPARP positive proportion (left) and the calculated Bliss synergy for that cPARP level (right). (F) Graphical representation of drug combinations (drug pairs connected by an edge) that showed a significant unexpectedly high cPARP over a predicted level at the given cell count. Node size indicates the number of drug pairs that the given drug appears with other drugs on the “unexpectedly apoptotic” list. Edge color indicates the drug pair concentration (standard or low) where the elevated cPARP was found; edge pattern indicates whether the elevated cPARP was found in the setting of low cell count or normal cell count (> 80% control), with elevated cPARP in the setting of normal viability potentially representing “slow” death kinetics for that combination.

To gain insight into the landscape of clinically relevant synergistic combinations targeted agents in cancer, we assembled a library of 108 compounds. Since we were interested in finding drug combinations with potential for clinical translation and for which mechanism of action would be tractable, we selected well-characterized oncology drugs approved by the Food and Drug Administration (FDA) or in late clinical trials; two-thirds of these agents have been in clinical use (Fig 1A and S1 Table). We then selected the most promising signal transduction inhibitors in clinical development and those that provided a large diversity of molecular targets to broadly cover cancer signaling pathways; this category constituted the vast majority of our library, at 67/108 drugs. We complemented these with drugs targeting cell cycle regulators, epigenetic modulators, nuclear hormone receptors, and other novel mechanisms under intense pre-clinical investigation. Finally, we included a limited number of drugs representative of major traditional cytotoxic chemotherapies. This drug panel expands substantially beyond a recently reported combination screen in melanoma utilizing 40 drugs [10]. A panel of 36 melanoma cells lines was selected representing major genotypic classes (S2 Table) and included six novel patient-derived short-term melanoma cultures (<10 passages from biopsy). Of the 30 previously established cell lines 19 have been characterized at the genomic level in further detail (S2 Table). Overall, this screen addresses a much larger number of drug combinations and cell lines than previously reported [9, 10, 12]. Given practical constraints of screening large numbers of combinations across full dose matrices of combinations, we selected two fixed-ratio dose combinations. We performed a screen run-in across ten cell lines to choose doses which resulted in > 70% viability to capture synthetic lethal events (data not shown). We then built a library of all 5,778 combinations corresponding to all possible two-drug combinations across the 108 drugs, and single drugs. We screened in 1,536-well ultra-high-throughput format using high-content and automated image analysis-based readout of cell count by nuclear staining (Fig 1B). In addition to cell counting by nuclear stain, we also systematically mapped drug combination effects on cell death by simultaneously using antibodies against cleaved PARP and normalizing to total nuclear count to obtain a cell death score. In total, for all drug combinations at two concentrations across all cell lines and measuring both cell count and cell death, we generated a landscape of >800,000 combinatorial drug data points (S3 Table). Given our limited coverage of the drug-drug dose matrix, we used the Bliss independence metric of synergy to represent unexpected combination effects [13–15].

Initial analysis of the synergy values showed that a large number of combinations demonstrated > 40% Bliss independence in more than one cell line, including several drugs with broad lethality and synergy when paired with any other drug across the cell line collection (Fig 1C). However, only 0.3% of synergistic combinations demonstrated true synthetic lethality, where individual drugs had no effect (> 80% viability) but strong synergistic viability < 50%. Thus, most drugs with synergistic interactions show some measureable effect of at least one single agent. We observed that some drugs sensitized cells to many other drugs; these included the proteasome inhibitor bortezomib, the pro-apoptotic B-cell lymphoma 2 (BCL2) family member inhibitor ABT263, and the microtubule inhibitor vincristine (Fig 1C). Interestingly, the majority of drug combinations showing synergy showed such synergy in three or fewer lines (56%; Fig 1D). Thus, most drug combination effects depend strongly on the cellular context. Even combinations showing profound synergies in a subset of cell lines are not synergistic or effective in other cell lines. Interestingly, this is true even for combinations that would be expected to be very broadly synergistic because they target two mechanistically complementary cellular processes. For example the combination of gemcitabine, a DNA damaging agent, together with the DNA damage repair pathway kinase CHK1 inhibitor AZD-7762, is strongly synergistic in only a subset (6/36) of cell lines. While this could be due to differences in DNA damage rate across cell lines, we observed multiple other cases of strong synergies that are seen only in a few lines.

A number of drugs induced increased cell death as measured by cPARP, including ABT263 and the broad kinase inhibitor midostaurin (Fig 1E). Fewer drugs shows broad synergy in cPARP induction compared to cell count; one exception was nilutamide, an androgen receptor antagonist. In general, drug-drug combinations reducing cell count after 72 hours also showed an increase in percentage of cells positive for cPARP staining (“fast” kinetics). However, a subset of combinations increased cPARP without affecting cell count (S1A and S1B Fig). We hypothesize that this subset likely represents combinations with “slow” kinetics of cell death induction. We developed a regression model for this relationship to identify these outlier apoptosis-inducing combinations in each cell line. Overall, 4% combinations showed an excess increased cPARP relative to cell count. Specific drugs induced apoptosis unexpectedly more often in this set of combinations (Fig 1F). Not surprisingly, ABT263 induced high cPARP broadly relative to cell count and was enriched in both “fast” and “slow” kinetic patterns combinations. Interestingly, fingolimod, a partial agonist of SP1R, a receptor thought to be involved in apoptotic cell recognition by immune cells rather than in cell autonomous regulation of apoptosis and bortezomib showed a “fast” pattern of death induction. In contrast, FK866, a nicotinamide phosphoribosyltransferase inhibitor showed a “slow” pattern of apoptosis induction.

### Drug synergy and off-target effects

To prioritize synergistic combinations for further mechanistic dissection, we first analyzed drug-drug interactions with the strongest Bliss independence scores across the most number of cell lines. Several of these interactions have been previously described in the literature, validating the screen in identifying *bona fide* synergistic interactions. Among these included MK1775, a WEE-1 inhibitor, and AZD7762, a CHK1/2 inhibitor (S2A Fig); dual inhibition of both cell cycle checkpoint kinases have been previously described in multiple tumor types in cell culture and *in vivo* [16]. Synergistic interaction between targeted inhibitors and BCL2 family member inhibition has been previously described [17, 18] and we also observed such an interaction between high doses of CHIR265, a pan-Raf inhibitor, and ABT263 (S2B Fig). ABT263 also sensitized a large number of cell lines (>10) in the primary screen to bortezomib and the phytochemical indole-3-carbinol. A particularly strong synergistic interaction was seen between BI78D3, a JNK inhibitor and TZDZ8, a GSK3β inhibitor (S2C Fig). However, we were unable to observe synergy by RNAi knockdown of either target class together with drug treatment (S2D Fig) or between an expanded collection of other JNK and GSK3β tool compounds (S2E Fig); furthermore, this synergy was seen across a range of other transformed and non-transformed cells (S2F Fig), suggesting this was a broad, idiosyncratic, and off-target cytotoxic synergistic interaction unlikely to be clinically useful due to its activity against non-transformed cells.

**Fig 2:**
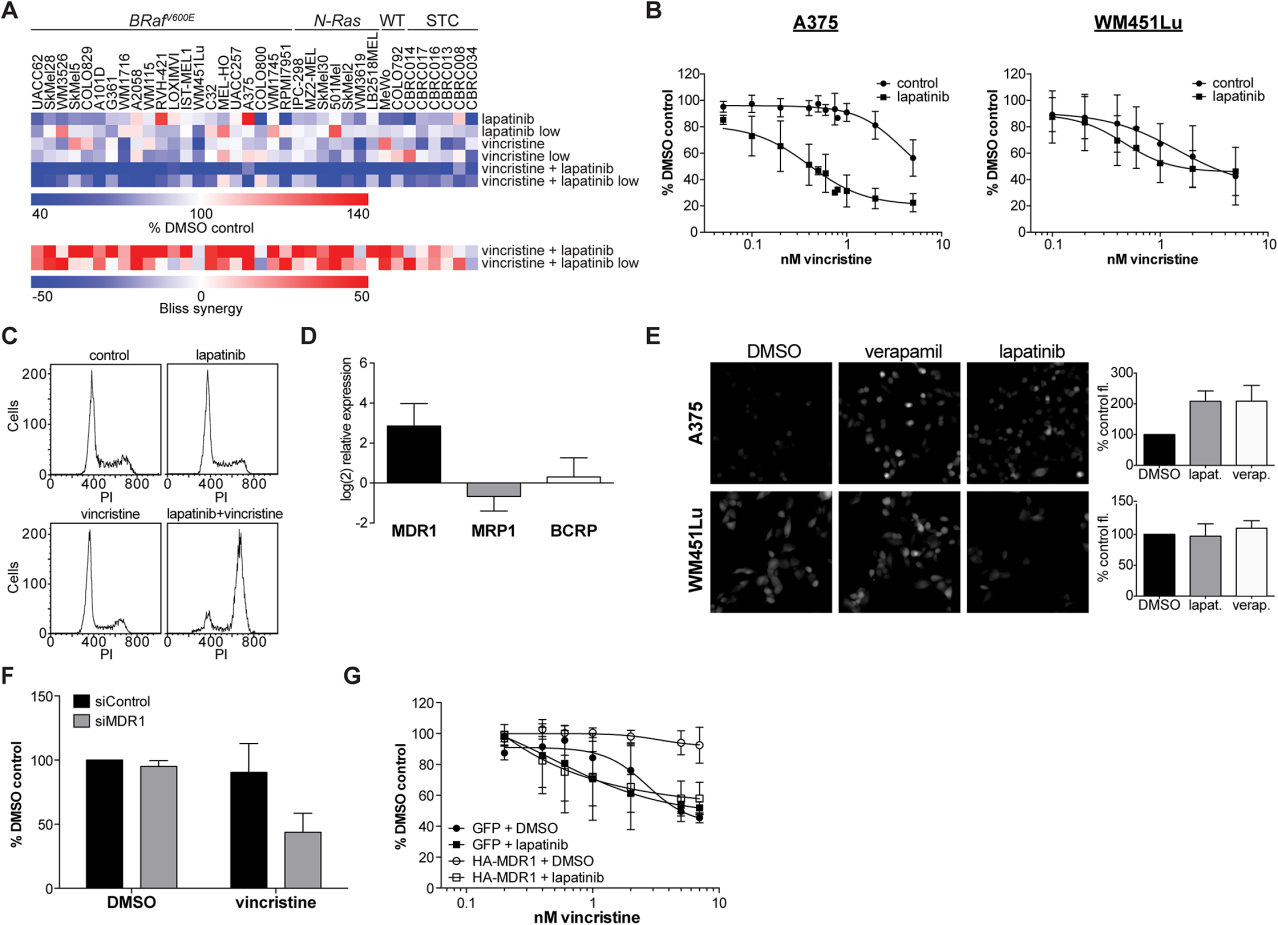
Cytotoxic potentiation by a MDR inhibitor. (A) Screening results showing the effects at both concentrations of vincristine and lapatinib, individually, and as a combination, showing strong synergy across most melanoma cell lines as indicated by high Bliss synergy scores. (B) Confirmation of the synergistic effect of the combination of lapatinib (5μM) in A375 (*n* = 19) but not WM451Lu (*n* = 14) cells. Error bars represent s.d. of measurement replicates. (C) Representative flow cytometry data showing lapatinib potentiates G_2_/M shift of A375 cell population consistent with increased vincristine effect. (D) Log_2_ relative expression of the given multi-drug resistance transporter in A375 versus WM451Lu cells, showing increased MDR1 expression in A375 cells. Error bars represent s.d. of measurement replicates (*n* = 9). (E) Calcein dye flux experiments showing increased fluorescence intensity (indicating decreased MDR flux) in the presence of lapatinib or control MDR inhibitor verapamil (both at 5μM); quantitation of cell grey values shown at left. Error bars represent s.d. of measurement replicates (*n* = 4, > 200 cells per replicate). (F) MDR1 knockdown by siRNA causes a synergistic effect on cell viability in the presence of 5nM vincristine. Error bars represent s.d. of measurement replicates (*n* = 7). (G) Overexpression of MDR1 (compared to GFP control) in WM451Lu decreases sensitivity to vincristine, an effect reversible with 5μM lapatinib. Error bars represent s.d. of measurement replicates (*n* = 4)

We selected one of these strong synergistic interactions for further mechanistic investigation. We observed a strong interaction between lapatinib, an EGFR family inhibitor, and vincristine, a microtubule inhibitor (Fig 2A). Vincristine is rarely used in melanoma therapeutic regimens and EGFR family members are not known to have a driver role in melanomas except in adaptive resistance to BRAF inhibitors [7]. We confirmed strong ~10X sensitization of some, but not all, melanoma cells to vincristine with lapatinib, with a Bliss value > 50% and Combination Index (CI) of 0.37 [19] (Fig 2B and S3A and S3B Fig), and several other EGFR family member inhibitors including erlotinib (S3C Fig). However, we were unable to sensitize A375 cells to vincristine following single or combinatorial knockdown of lapatinib targets EGFR and HER2 (S3D Fig). Furthermore, cell cycle analysis showed synergistic arrest in G_2_/M with the vincristine-lapatinib combination, as would be expected with increasing vincristine dose (Fig 2C). Lapatinib and other 4-anilinoquinazoline-derived tyrosine kinase inhibitors have been described as inhibitors of the P-gp family of multidrug resistance (MDR) transporters [20, 21]. Verapamil, a canonical MDR1 inhibitor, resulted in a similar synergistic interaction with vincristine (S3E Fig). Given the strong synergy between vincristine and lapatinib in A375 but not WM451Lu cells, we investigated whether differential MDR family expression may underlie the cell line specificity of the synergistic interaction. Overall, we observed a general trend towards increased synergy between vincristine and lapatinib in cell lines with higher MDR1 mRNA expression. (S3F Fig). Because individual cell context may influence the robustness of the general trend, we specifically compared a sensitive to an insensitive cell line. We observed >8-fold expression of MDR1 mRNA in A375 cells vs. WM451Lu cells (Fig 2D). Furthermore, lapatinib increased retention of a MDR substrate dye similarly to verapamil (Fig 2E). Knockdown of MDR1 by RNAi in A375 cells sensitized the cells to vincristine cell growth inhibition (Fig 2F and S3G Fig). Overexpression of MDR1 in WM451Lu cells induced resistance to vincristine in a lapatinib-dependent manner (Fig 2G and S3H Fig). Thus, despite the “targeted” nature of some kinase inhibitors, their activity against MDR family members can produce a synergistic effect unrelated to their primary targets. Consistent with this, we observed synergy between vincristine and lapatinib across a range of other rapidly proliferating cells (S3I Fig). Similar findings of promiscuous synergistic interactions via bioavailability have been found in yeast anti-fungal drug combination screens [22]. A large number of compounds across a range of structural classes can display MDR inhibition, supporting our observation that vincristine was sensitized by a large number of other drugs (Fig 1D).

### VEGFR/PDGFR antagonists synergize with BRAF inhibitors

**Fig 3:**
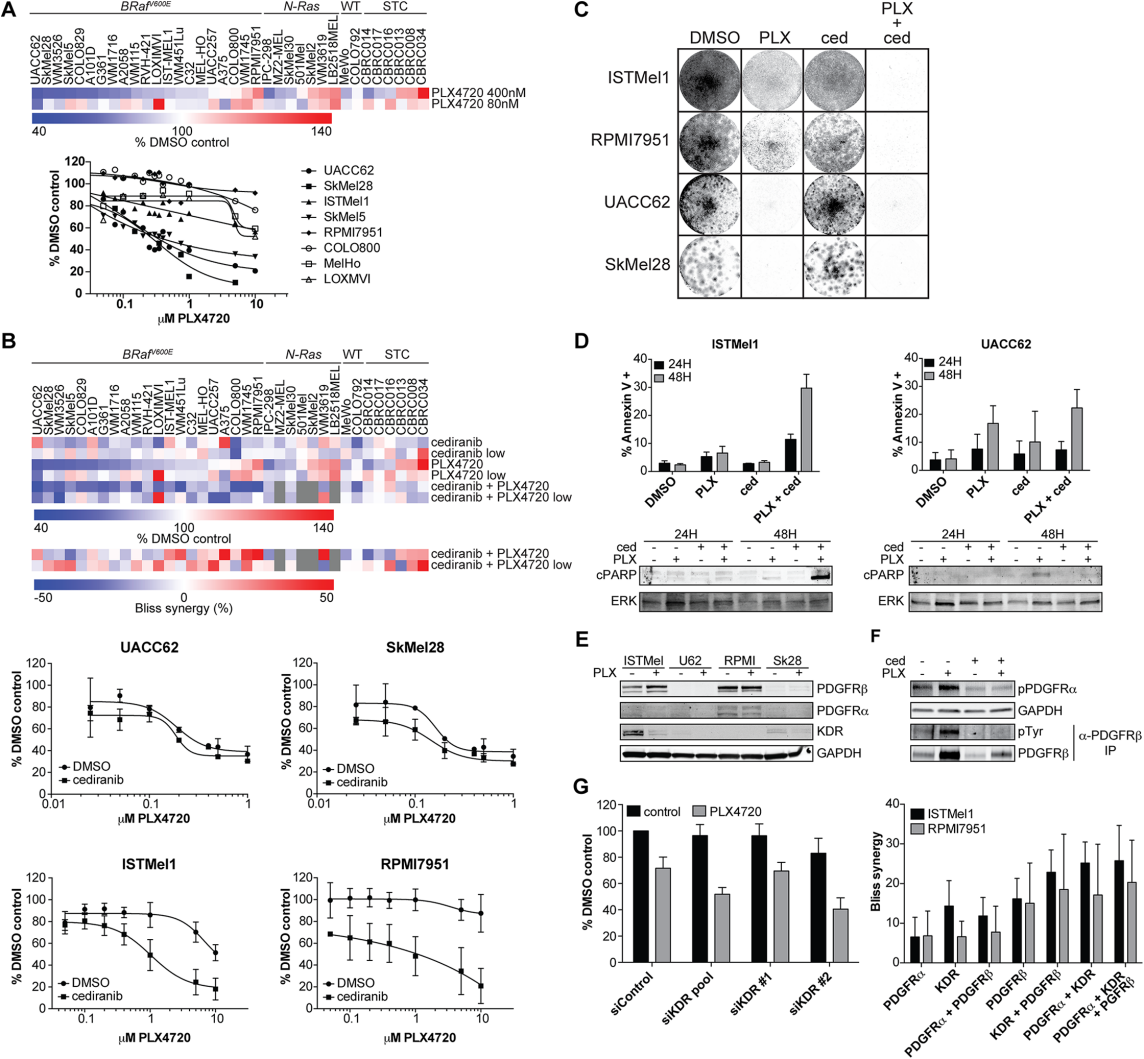
Cediranib and PLX4720 synergize in intrinsically PLX4720-resistant melanoma cells. (A) Effect of PLX4720 (PLX4720) across melanoma cells in the primary screen, demonstrating some *BRAF* mutant melanomas display intrinsic resistance to BRAF inhibition. Resistance (defined as > 50% viability at > 5μM PLX4720) in these lines was confirmed in secondary assays (below). (B) Cediranib displayed synergistic effects with PLX4720 in several intrinsically resistant lines in the primary screen; these effects were verified in standard growth assays in secondary screens (below, with 2μM cediranib). BRAF inhibitor-sensitive lines UACC62 and SkMel28 showed no synergy at any PLX4720 dose, while resistant ISTMel1 and RPMI7051 lines showed strong synergy with cediranib (Bliss > 30%). Error bars represent s.d. of measurement replicates (*n* = 3–4). (C) Long-term crystal violet-stained colony growth assays confirmed sustained synergy between PLX4720 (PLX) and cediranib (ced) in resistant ISTMel1 and RPMI7951 lines but not PLX4720 sensitive lines UACC62 and SkMel28. (D) Annexin V assays showed that cediranib induced apoptosis when combined with PLX4720 in resistant ISTMel1 cells, as compared to sensitive UACC62, and as confirmed by Western blotting to cleaved PARP (below). Error bars represent s.d. of measurement replicates (*n* = 3). (E) Western blotting confirmed expression of cediranib targets PDGFRβ, and variably, KDR and PDGFRα in PLX4720-insensitive ISTMel1 and RPMI7951 cells, with weaker or absent expression in PLX4720 sensitive UACC62 and SkMel28 cells. Twenty-four hour treatment with PLX4720 moderately suppressed KDR and induced PDGFRβ expression, a pattern also seen *in vivo* (S9A Fig). (F) Western blotting confirmed on-target suppression of PDGFRα and PDGFRβ phosphorylation (latter after immunoprecipitation and blotting to phospho-tyrosine given low abundance). KDR phosphorylation was too weak to be observed by Western routinely in culture but was seen *in vivo* (see S9A Fig). (G) RNAi-mediated knockdown of KDR (left) with pools or individual siRNAs showed synergy to ~30%; synergy was proportional to KDR knockdown (see S7A Fig). Error bars represent s.d. of measurement replicates (*n* = 6). At right, average Bliss values over multiple replicates showed increasing synergy with simultaneous knockdown of multiple cediranib targets KDR and PDGFRβ, and, more weakly, PDGFRα. Error bars represent s.d. of measurement replicates (*n* = 5–8).

To identify more specific synergistic combinations, we focused on combinations that might address intrinsic resistance to the BRAF inhibitor vemurafenib. We identified several BRAF^V600E^ cell lines, including ISTMel1 and RPMI7951, which displayed resistance to PLX4720 even at high doses (> 5μM, growth inhibition < 50%), when compared to sensitive lines such as UACC62 and SkMel28 (IC_50_ < 500nM) (Fig 3A). Many of the 107 other drugs in our library showed synergistic interactions with PLX4720 in specific cell contexts (S4A Fig). We used the statistical analysis of microarray (SAM) approach [23] to identify combinations with PLX4720 which were specifically synergistic in the PLX4720-resistant cell lines (S4B Fig). Among these included the HDAC inhibitor vorinostat, recently found to synergize with BRAF inhibitors in some melanoma cell lines [24]. We noted significant synergy between the RTK inhibitor cediranib and PLX4720 in these resistant cell lines (Fig 3B), with a CI of 0.35 (S5A Fig), but not in sensitive lines, even at low doses of PLX4720. Synergy was also observed between cediranib with the MEK inhibitor selumetinib (AZD6244), and with the triple combination of cediranib, PLX4720, and selumetinib, suggesting a general interaction between cediranib and inhibition of BRAF-driven MAPK signaling (S5B and S5C Fig). Long-term growth assays confirmed a strong synergistic interaction (Fig 3C). The combination of cediranib and PLX4720 rapidly induced apoptosis in resistant cells, as shown by Annexin V staining and cPARP Western blotting (Fig 3D). While single agent PLX4720 treatment caused cell cycle arrest in sensitive cell lines, there was no significant effect of the drug combination on cell cycle (S5D Fig). In contrast to the results with lapatinib, we found no sensitization of resistant lines to PLX4720 with the MDR inhibitor verapamil (S5E Fig).

Cediranib is a potent and selective inhibitor of the PDGFR and VEGFR family of receptor tyrosine kinases in clinical trials, used primarily as an anti-angiogenesis agent [25]. While PDGFRα and β overexpression previously has been linked to acquired vemurafenib resistance [6, 7, 26], this family has not been implicated in cell-autonomous primary vemurafenib resistance. To further understand the mechanism of the drug combination, we tested an expanded list of RTK inhibitors currently in clinical use or development. Some, but not all, similar inhibitors showed synergies with PLX4720, and in some but not all resistant lines (S6A Fig). Interestingly, only cediranib and tivozanib, not the more specific inhibitors axitinib (VEGFR) or crenolanib (PDGFR), showed consistent and potent synergistic activity with PLX4720 across a wide range of doses, suggesting a specific inhibitory activity of these compounds on a subset of targets. Cell line expression databases showed expression of cediranib targets in resistant cell lines (S6B Fig), which we confirmed by Western blotting (Fig 3E). In general, we also found that melanoma cell lines express higher levels of KDR than any other lineage in a large collection of cancer cell lines (S6C Fig), suggesting a specific cell-autonomous role for KDR in melanoma development. We detected on-target suppression by cediranib of phosphorylation of both PDGFRα and PDGFRβ (Fig 3F). Next, we attempted to recapitulate synergy between PLX4720 and cediranib by knockdown of its primary targets. siRNA knockdown of KDR showed a synergistic decrease in cell number when combined with PLX4720 treatment proportional to knockdown efficiency; furthermore, simultaneous knockdown of multiple cediranib targets including PDGFRα and PDGFRβ increased synergy with BRAF inhibition (Fig 3G and S7A and S7B Fig). Although we cannot exclude the possibility that some of the synergistic activity is due to inhibition of other RTK targets not included in our analysis, these results suggest that the particularly strong synergistic activity of cediranib in combination with PLX4720 is due to inhibition of KDR and related RTKs including PDGFRα and PDGFRβ.

Activation or re-activation of major pathways downstream of RTKs such as mitogen-activated protein kinase (MAPK) and phosphatidylinositol-3-OH kinase (PI(3)K)–AKT pathways previously has been implicated in resistance to targeted therapies including vemurafenib in both *in vitro* and clinical studies [8, 27–32]. Unlike most studies of resistant cell lines, PLX4720 completely suppressed ERK activation in the intrinsically-resistant lines; furthermore, dual inhibition of MAPK pathway with PLX4720 and selumetinib showed no synergistic interaction (S8A and S8B Fig), and cediranib continued to show synergy in triple combination with both PLX4720 and selumetinib (S5C Fig). These results suggested additional pathways beyond MAPK are related to cediranib’s activity. Cediranib suppressed S6K phosphorylation in both sensitive (UACC62) and resistant (ISTMel1) lines, and the combination suppressed activation in resistant lines of most Akt pathway components by pathway phospho-antibody arrays (S8C Fig). To determine whether this was simply a marker of RTK inhibition and/or synergy, or the mechanism of the observed synergy, we tested whether PI3K/Akt pathway inhibitors could synergize with PLX4720. Interestingly, Akt pathway inhibition showed only variable and moderate synergy with PLX4720 despite strong pathway suppression (S8D and S8E Fig). These results suggest that suppression of MAPK or PI3K signaling downstream of KDR/PDGFR RTKs only partially contributes to synergy between cediranib and PLX4720 and therapeutically tractable intrinsic resistance mechanisms extend beyond these two pathways. Among potential downstream pathway affected by the PI3K/Akt pathway suppression observed by the drug combination, β-catenin is a known substrate of GSK3β, whose activation may follow the decreased Ser9 phosphorylation observed with the combination. Although several β-catenin pathway modulators showed no antagonism or synergy with PLX4720 in our primary screen (S9A Fig), and GSK3β has a number of known substrates [33], this pathway may be one of the potential effectors of the drug combination.

**Fig 4:**
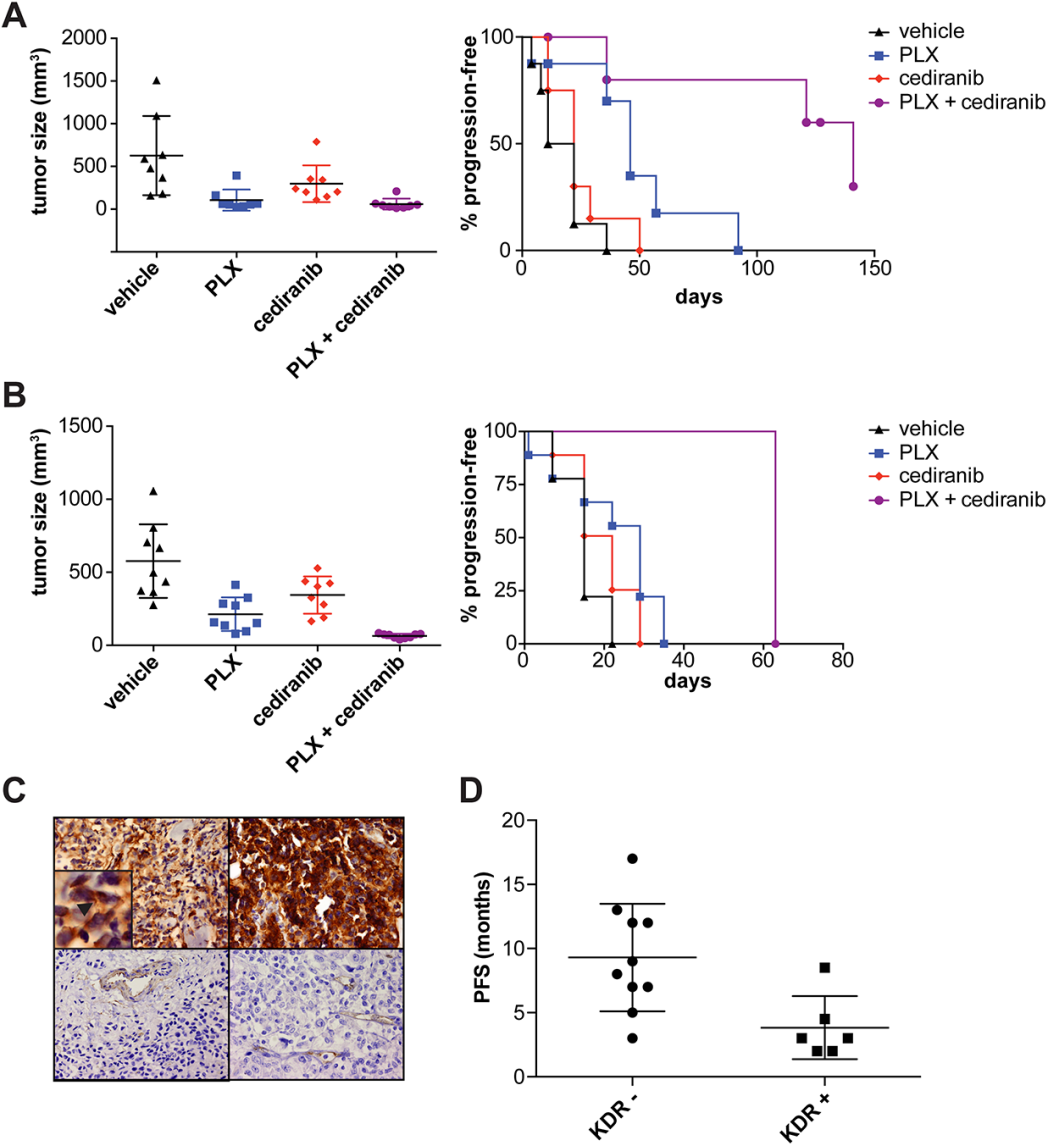
Cediranib synergizes with PLX4720 *in vivo*. (A) ISTMel1 xenografts were generated in immunodeficient mice (*n* = 8 per group), which were treated with PLX4720 or control chow and given cediranib or water by oral gavage. ISTMel1 xenografts showed an initial response to PLX4720 treatment alone after approximately two weeks, with some additional but non-significant response to cediranib treatment. Values are shown as mean +/- S.E.M. ranges, with significant differences in mean tumor size of both PLX4720 and PLX4720 and cediranib-treated mice compared to control treatment by ANOVA. However, the combination significantly delayed progression (defined as > 500 mm^3^ size) beyond this initial response (right, *p* < 0.002 between PLX4720 and PLX4720 + cediranib arms by log-rank test). (B) In contrast to ISTMel1 xenografts, RPMI7951 xenografts showed a significant difference by ANOVA test in initial response to PLX4720 versus PLX4720 and cediranib treatment after three weeks, and, right, showed a prolonged delay of progression (defined as tumor > 250 mm^3^) of completely PLX4720-resistant tumors (*p* < 0.0001 between PLX4720 and PLX4720 and cediranib arms by log-rank test). (C) KDR staining of tumor biopsies from patients entering clinical trials for BRAF with or without MEK inhibitors demonstrated some had strong membrane KDR staining (top) throughout the tumor (inset showing membrane staining, at arrowhead), while others were negative for KDR staining (bottom) except for expected endothelial staining. (D) Comparison of progression-free survival of patients with (*n* = 6) and without (*n* = 10) membrane KDR staining showed a significant reduction in PFS (9.3 vs. 3.8 months, *p* < 0.01 by Student’s t-test) if the patient’s biopsy expressed KDR.

Next, we tested whether synergistic inhibition of growth of melanoma cells by cediranib and PLX4720 could be recapitulated *in vivo*. In two xenograft models with the resistant lines ISTMel1 and RPMI7951, we observed a strong interaction between the inhibitors on tumor progression (Fig 4A and S9A–S9C Fig). ISTMel1 showed moderate initial sensitivity to PLX4720, but cediranib suppressed later growth of tumors while on PLX4720; in contrast, in RPMI7951, the combination of cediranib and vemurafenib decreased initial tumor growth beyond the effect of either drug alone.

Finally, we tested whether expression of any targets of cediranib could predict initial responses of patients receiving BRAF or MEK-directed therapy (S4 Table). We found high expression of tumor cell (as opposed to endothelial or stromal cell) KDR in a number of melanoma patient samples by immunohistochemistry (Fig 4C). Patients with high levels of membrane KDR staining showed decreased progression free survival when on BRAF and/or MEK-directed therapy (Fig 4D); PDGFRβ staining provided no predictive value (data not shown).

## Discussion

As more cancer patients receive driver oncogene-directed therapy, there is greater recognition of the need for combination therapies that can induce more complete initial responses and prevent development of acquired resistance. Indeed, the small subpopulation of patients who achieve complete responses to BRAF and BRAF/MEK inhibition have the most durable responses [34, 35]. Unbiased combinatorial drug screening of targeted therapies in clinical development can identify novel synergistic interactions that are rapidly clinically actionable. Such screening also capitalizes on kinase inhibitor polypharmacology to identify resistance “programs” due to activation of multiple targets [36]. We describe here an example of the output from such a large screen and a novel statistical framework to reduce singlet noise in the data that is likely generalizable to other large combinatorial datasets. In general, we found that broadly synergistic drug-pairs can display non-specific or off-target toxicity in rapidly dividing cancer types and occasionally in non-transformed cells; most displayed a pattern of IC_50_ leftward shifts in their activity rather than *E*_*max*_ shifts. In contrast, we identified a specific synergy with VEGFR/PDGFR inhibitors that display an *E*_*max*_ shift in PLX4720-resistant cells. A recent study has shown that shallow *E*_*max*_ responses can be due to increased cell to cell variability and may be equally important as IC_50_ in judging single-agent drug responses [37]. Synergistic interactions that affect specifically maximum effect (*E*_*max*_; “effect boost” [38]) may be more relevant to development of targeted therapy combinations.

Our results suggest that KDR may be a predictive marker of vemurafenib and or MEK inhibitor response and the combination of cediranib and BRAF or MEK inhibitors are an actionable combinatorial regimen for melanoma patients with BRAF mutations. Interestingly, analysis of KDR alterations as a general prognostic marker in melanoma using The Cancer Genome Atlas (TCGA) data through the C-Bio portal [39] did not show a statistically significant correlation to overall or disease-free survival, suggesting KDR becomes an important marker only in the setting of BRAF-directed MAPK pathway therapy. Previous studies have shown additive effects of cediranib with a range of other targeted and traditional cytotoxic drugs including selumetinib *in vivo* in several non-melanoma tumor types [40, 41]; these effects were attributed to non-autonomous effects on tumor vasculature. Here, we describe a cell-autonomous effect of cediranib in combination with MAPK signaling suppression specifically in BRAF^V600E^ mutant but PLX4720-resistant cells. There is, however, growing recognition of a cell-autonomous role for VEGF receptors in tumor cells [42]. Indeed, a trial of cediranib and selumetinib has recently been initiated in solid tumors (NCT01364051) but may be more effective if directed towards melanoma patients with both BRAF^V600E^ mutation and elevated KDR/PDGFRα/β expression. Furthermore, we found synergistic interaction between cediranib and PLX4720 in non-melanoma BRAF^V600E^ mutant cell lines with expression of KDR/PDGFRα/β family members (S9D Fig), suggesting the combination may be effective broadly across BRAF^V600E^ mutant cancers beyond melanoma.

## Materials and Methods

### Cell lines

Cells were grown in RPMI or DMEM medium supplemented with 10% FBS and 1% penicillin/streptomycin and 2 mM L-glutamine, and maintained at 37°C in a humidified atmosphere at 5% CO_2_. “WM” lines were obtained from cryopreserved collections at the Wistar Institute, courtesy of M. Herlyn. “CBRC” lines were derived from biopsies of MGH melanoma patients, as described [43]. All were screened and archived < 10 passages from biopsy. All other cell lines were derived from the Center for Molecular Therapeutics (CMT) cell line collection. As previously described [11], all CMT cell lines have been short tandem repeat (STR) verified and confirmed to be mycoplasma-free; genotyping for the most frequently mutated genes was performed by sequencing to base-pair resolution across all coding exons for each gene by capillary sequencing; and for gene expression analysis, Trizol-extracted RNA was hybridized to the HT-HGU122A Affymetrix array, and normalized gene expression intensities were generated using the Robust Multi-Array Average (RMA) algorithm. Additional cell line characterization for other cell lines indicated in S2 Table was previously described by others [44], as follows: BRAF, NRAS, and PTEN were sequenced by standard Sanger sequencing, and Trizol-extracted RNA was hybridized to the HT-HGU133A Affymetrix array, followed by analysis using MAS 5.0. More information on the cell lines, including their SNP and STR profiles, can be found on the Genomics of Drug Sensitivity in Cancer website (http://www.cancerRxgene.org).

### Compounds, antibodies and reagents

Compounds for screening were purchased from the commercial vendors as listed in S1 Table. Other compounds were purchased from commercial vendors as follows: verapamil (VWR #89152–492), CHIR-99021 (Thermo Fisher Scientific #508306), SB-216763 (Thermo Fisher Scientific #508408), SP600125 (LC Laboratories #S-7979), CC-401 (Synkinase #SYN-1028), tivozanib (AV951, Selleck #S1207), crenolanib (Selleck #S2730), axitinib (Selleck #S1005), foretinib (Selleck #S1111), vandetanib (LC Laboratories #V-9402), PDGFRi III (Santa Cruz Biotechnology #sc-204173), PDGFRi IV (Santa Cruz Biotechnology #sc-205794), and PDGFRi V (EMD Biosciences #521234). Antibodies were obtained from commercial vendors as follows: cleaved PARP (Cell Signaling Technology #9541), ERK (Cell Signaling Technologies #9102), dpERK (Cell Signaling Technologies #9106), PDGFRβ (Cell Signaling Technologies #3169), PDGFRα (Cell Signaling Technologies #5241), phospho-PDGFRα (Tyr762, Cell Signaling Technologies #12022), GAPDH (Cell Signaling Technologies #2118), KDR (Cell Signaling Technologies #2479), HA (Cell Signaling Technology #2367), MDR1 (Abcam # ab129450), phospho-p70 S6K (Thr421/Ser424, Cell Signaling Technologies #9204), phospho-Akt (Ser473, Cell Signaling Technologies #4058), phospho-Tyrosine (Cell Signaling Technologies #9411), and Annexin V (BD Biosciences #556547). PathScan Akt Signaling Antibody Array Kit (Cell Signaling Technologies #9700) was used to measure pathway activity following drug treatment. CellTiter-Glo Viability Assay was from Promega (G7571).

### Ultra high-throughput combinatorial drug screen

Approximately 250 cells per well for each cell line were seeded in each well of 1,536-well microtiter plates (PerkinElmer) and incubated overnight. Combinatorial drug library was prepared as 300X stocks in 384 well plate and 20nL pin-transferred to 1,536-well plates. Plates were incubated for 72 hours and fixed in 4% formaldehyde, washed in PBS containing 0.1% Triton, and incubated overnight with antibodies (1:500) to cleaved PARP (above). The plates were then washed twice, incubated with Alexa 647 secondary antibodies (1:1000, Life Technologies) and DAPI 2–16 hours, and washed with PBST. Plates were then imaged using the Cellworx high-throughput microscope (Applied Precision Inc.) and counting nuclei and cells with cPARP staining with the Multi-Wavelength Cell Scoring module of the MetaExpress image analysis software (Molecular Devices). Z’ values were between 0.8–0.9 for most cell lines.

### Cleaved PARP data analysis

For every cell-line in the collection, we performed a dual regression fit (exponential and linear) on the relationship between cell counts (% viability) and apoptosis (% cPARP) levels, using the Matlab^R^ toolbox ‘ezyfit’:

- exponential curve fitting approach: evaluate ‘a’ and ‘*τ*’, within $f(x)\;=\;a\;*\;e^{-\frac{x}{\tau }}$.
- linear fitting approach: evaluate ‘a’ and ‘*τ*’, within *f*(*x*) = *a* * *x* + *τ*.

Choosing the best fit in terms of exponential or linear models was based on the Pearson correlation between the measured compound combinations (*f*(*x*)) and their fitted values ($\hat{f}(x)$). Furthermore, statistically significant cPARP values were selected using a two-sample t-test, performed on the residuals from the above-mentioned selected curve fit. The most significant tested combinations that resulted in abnormally increased cPARP values (“unexpectedly apoptotic”), for viability measures within low and/or normal cell counts are graphically depicted in Fig 1F.

We observed significant variability in the level of cleaved PARP on a cell line to cell line basis, with some cell lines displaying much higher basal and induced cPARP relative to other cell lines. This variability may be due to technical or biological differences.

### Plasmids and siRNA

Pooled siRNAs targeting JNK1 (Dharmacon, #L-003514-00-0005), JNK2 (Dharmacon #L-003505-00-0005), JNK3 (Dharmacon #L-004324-00-0005), EGFR (Dharmacon L-003114-00-0005), HER2 (Dharmacon #L-003126-00-0005), KDR (Dharmacon #L-003148-00-0005), PDGFRα, PDGFRβ (Dharmacon #L-003163-00-0005), MDR1 (Dharmacon #L-003868-00-0005), GSK3β (Dharmacon #L-003010-00-0005) or control non-targeting siRNA (Dharmacon #D-001810-10-20) or individual siRNAs targeting KDR (Dharmacon #LU-003148-00-0002; siKDR #1 is J-003148-09; siKDR #2 is J-003148-11) were transfected at final concentration of 10nM (single gene knockdown) or 20nM (combinatorial gene knockdown) using the lipidoid delivery agent C12-113-B as previously described [17]. Lipidoid was dissolved in 25mM NaOAc buffer (pH~5.2) and added to solution of siRNA for complexation.

### Cell viability

All secondary viability assays were performed using the CellTiter Glo assay (Promega) after seeding 3,000 cells per plate, and the following day adding the indicated drugs for 96 hours. For long-term cell proliferation assays, cells were seeded into 12-well plates and cultured both in presence of drugs or controls as indicated, and stained by crystal violet staining.

### RT-PCR

mRNA was extracted from cell lines using the RNeasy kit (Qiagen) and homogenized using the Qiashredder kit (Qiagen). Total mRNA was used for subsequent reverse transcription using the Kapa SYBR FAST qPCR master mix (Kapa Biosystems). Expression was normalized to *actB* or *hTBP* mRNA as indicated. Primers were as follows, all 5’ to 3’ orientation: hTBP (forward), AACAACAGCCTGCCACCTTA, (reverse), GCCATAAGGCATCATTGGAC; *actB* (forward) CTGGAACGGTGAAGGTGACA, (reverse), AAGGGACTTCCTGTAACAATGCA; *EGFR* (forward), AGGCACGAGTAACAAGCTCAC, (reverse), ATGAGGACATAACCAGCCACC; *HER2* (forward), GCTCATCGCTCACAACCAAGT, (reverse), ACAGGGGTGGTATTGTTCAGC; *JNK1* (forward), TGTGTGGAATCAAGCACCTTC, (reverse), AGGCGTCATCATAAAACTCGTTC; *JNK2* (forward), GAAACTAAGCCGTCCTTTTCAGA, (reverse), TCCAGCTCCATGTGAATAACCT; *JNK3* (forward), CAAATGTTGTGTGGCATTAAGCA, (reverse), TCAATGTGCAATCAGACTTGACT; *GSK3β* (forward) CCACTGTTGTCACCTTGCTG, (reverse), GAGTGATCATGTCAGGGCG; *KDR* (forward), GGCCCAATAATCAGAGTGGCA, (reverse), CCAGTGTCATTTCCGATCACTTT; *PDGFRα* (forward), TGGCAGTACCCCATGTCTGAA, (reverse), CCAAGACCGTCACAAAAAGGC; *PDGFRβ* (forward), AGCACCTTCGTTCTGACCTG, (reverse), TATTCTCCCGTGTCTAGCCCA; *MDR1* (forward), TTGCTGCTTACATTCAGGTTTCA, (reverse), AGCCTATCTCCTGTCGCATTA; *MRP1* (forward), CGACATGACCGAGGCTACATT, (reverse), AGCAGACGATCCACAGCAAAA; *BCRP* (forward), ACGAACGGATTAACAGGGTCA, (reverse), CTCCAGACACACCACGGAT.

### Western Blotting and Immunohistochemistry

For western blotting, whole cell lysates were collected in Radio-Immunoprecipitation Assay (RIPA) lysis buffer supplemented with protease and phosphatase inhibitors (Roche). Centrifuged supernatants normalized for protein content by bicinchoninic acid (BCA) protocol (Pierce). Equal amounts of protein were resolved by electrophoresis on 4–15% or 10–20% gradient gels and transferred to polyvinylidene difluoride membranes. IRDye 800CW (Rockland) or Alexa 680-conjugated secondary antibodies were used, followed by imaging on the LiCor Odyssey (Li-Cor Biosciences). For immunohistochemistry, 4 μM sections of formalin-fixed, paraffin-embedded specimens were heated at 60°C, deparaffinized in xylene, and hydrated in a series of ethanol dilutions. Epitope retrieval was by microwaving (5 min at 850w, 15 min at 150w) in 10 mM Tris-EDTA buffer pH 9.0. Slides were blocked 10 minutes in 3% BSA in TBST (Tris pH 7.6, 0.05% Tween-20). Primary antibodies were as follows: KDR, 1:100 in 3% BSA in TBST, clone 55B11 (Cell Signaling #2479S); PDGFRβ, 1:100 in 3% BSA in TBST, clone 28E1 (Cell Signaling #3169S). Slides underwent 10 min peroxidase block in 3% H_2_O_2_. Secondary antibody Dako EnVision anti-rabbit (K4003, ready-to-use) was applied for 30 minutes. Slides were developed with DAB+ (Dako K3468) for 10 min, and counterstained 1 min with hematoxylin (Vector H-3401), prior to dehydration and mounting. Slides were imaged on an Olympus BX51 microscope with Olympus DP25 camera using Olympus WHN10X-H/22 oculars, Olympus UPlan FL N -20x/0.50 and -40x/0.50 objectives, an Olympus DP25 camera, and images acquired using Olympus DP2-TWAIN software and Adobe Photoshop 7.0. Slides were scored for intensity and distribution of KDR and PDGFRβ by a dermatopathologist blinded to clinical outcome. Scoring of staining (by A. P.) and bio statistical analysis was doubled-blinded.

### Xenografts

For mouse xenotransplant experiments, 1 × 10^7^ ISTMel1 or RPMI7951 cells were injected subcutaneously into the flanks of female *nu/nu* (ISTMel1) or NSG (RPMI7951) female mice aged to 7–8 weeks. Six to eight animals were used per experiment as per standard procedures. After tumors reached 100–150 mm^3^ in size, animals were assigned to treatment groups by simple randomization and given *ad libitum* mouse chow containing 2% PLX4720 by weight or control chow acquired from Plexxikon Inc. Cediranib (6mg/kg) was administered daily by oral gavage 5 days per week. Tumor volume was calculated by the formula ½ x (length x width^2^); investigator was not blinded to treatment group. All studies and procedures involving animal subjects were approved by the Institutional Animal Care and Use Committees of Massachusetts General Hospital and Dana-Farber Harvard Cancer Center and were conducted strictly in accordance with the approved animal handling protocol.

### Melanoma patient tumor samples

De-identified biopsies of patients for generation of short-term cultures or for immunohistochemical staining to KDR or PDGFRβ were obtained after informed consent on Dana-Farber/Harvard Cancer Center-approved protocols 11–181 and 02–017. All samples were analyzed via hematoxylin and eosin (H&E) staining to confirm that viable tumor was present.

### Other statistical analyses

Cell culture growth data were modeled using a nonlinear sigmoid regression curve using GraphPad Prism 6 for Mac (GraphPad). All statistical tests excluding those explicitly discussed above were implemented using GraphPad. For animal ANOVA tests, the data were log transformed prior to analysis to equalize variance by Bartlett’s test; Tukey’s test was used to account for multiple comparisons. Student’s *t*-test (two-sided) of patient samples was implemented after confirming normal distribution and equal variance by *F* test. To identify significantly synergistic combinations with PLX4720 specific to the PLX4720-resistant cell lines, we performed SAM analysis on both the original and modeled combination data. Heat maps, hierarchical clustering, and SAM analysis were implemented using the MeV platform [45].

## Supporting Information

S1 Fig Combinatorial drug viability relationship with apoptosis. (A) Single drug results across all melanoma cell lines, plotted as the relationship between the normalized cell count (*x* axis) and the normalized and log-transformed percent of cPARP positive nuclei (*y* axis). Shown are the data for the low (left) and standard (right) library concentration. In general, single drugs that result in reduced viability also show an increase in cPARP percentage. Insets show population of single drugs resulting in relatively normal viability, some of which also show increase cPARP staining. (B) As in (A) but shown are results for all combinations across all melanoma cells lines. Also demonstrated in insets are specific population of drug-drug-cell line triads with relatively normal viability but increased cPARP staining.
(TIF)

S2 Fig Synergistic drug-drug interactions. (A) AZD7762, a CHK1/2 inhibitor, displayed broad synergistic effects with MK1775, a WEE1 inhibitor, across multiple melanoma cell lines. This effect was confirmed in secondary experiments, below, showing synergistic interaction in A375 cells. Error bars represent s.d. of measurement replicates (*n* = 4). (B) CHIR265, an inhibitor of BRAF and other kinases, showed a synergistic interaction with ABT263, a BCL2 family inhibitor, at high doses of CHIR265; this effect was confirmed (below) in UACC62 cells. Error bars represent s.d. of measurement replicates (*n* = 3). (C) BI78D3, a JNK family inhibitor, showed strong synergy with TZDZ8, a GSK3β inhibitor, across multiple melanoma cell lines. This interaction was confirmed in secondary experiments (below) in A375 cells. Error bars represent s.d. of measurement replicates (*n* = 8). (D) siRNA knockdown of TZDZ8 target GSK3β (top, *n* = 5) or BI78D3 targets JNK1, JNK2, or JNK3 (bottom, *n* = 3) showed no synergy with 500nM BI78D3 or 5μM TZDZ8, respectively. Error bars represent s.d. of measurement replicates. RT-PCR confirming siRNA-mediated target knockdown is shown at right. Expression is normalized to *ActB*. Error bars represent s.d. of measurement replicates (*n* = 2). (E) No synergy was observed between 5μM TZDZ8 and a variety of other JNK inhibitors, including CC401 (*n* = 2), SP600125 (*n* = 3), and TCS JNK5a (*n* = 2). No synergy was observed between BI78D3 and other GSK3β inhibitors, including 1μM CHIR99021 (*n* = 4) and 1μM SB216763 (*n* = 3). Error bars represent s.d. of measurement replicates. (F) Synergistic interaction was seen between TZDZ8 and 5μM BI78D3 across a range of non-melanoma cells, including BxPc3 pancreatic cell line (*n* = 2), DU145 prostate cell line (*n* = 2), MCF7 breast cancer cell line (*n* = 2), and normal human fetal melanocytes (*n* = 2). Summary of maximum Bliss measurements for each cell line is shown on bottom right. Error bars represent s.d. of measurement replicates.
(TIF)

S3 Fig Synergy between vincristine and lapatinib. (A) Isobologram demonstrating significant synergy between vincristine and lapatinib, as shown in A375 cells. Combination Index was, on average 0.37, with minimum of 0.085. (B) Confirmation of synergy between vincristine and 5μM lapatinib in multiple other melanoma cell lines, including UACC62 (29% Bliss, *n* = 7), SkMel30 (36% Bliss, *n* = 3), and IPC298 (47% Bliss, *n* = 4). Error bars represent s.d. of measurement replicates. (C) Significant synergy was also seen in the primary screen across multiple melanoma cell lines between vincristine and erlotinib. This synergy was confirmed in secondary experiments in A375 cells (right). Error bars represent s.d. of measurement replicates (*n* = 3). (D) siRNA-mediated knockdown of lapatinib targets EGFR and HER2 demonstrated no synergy with 2nM vincristine either alone (left, *n*_=_ 6) or in combination (right, *n* = 5). Error bars represent s.d. of measurement replicates. Target knockdown was confirmed by RT-PCR measurement and normalized to *hTBP* or *ActB* (below). Error bars represent s.d. of measurement replicates (*n* = 4). (E) Canonical MDR inhibitor verapamil (5μM) showed significant synergy with vincristine in A375 cells. Error bars represent s.d. of measurement replicates (*n* = 7). (F) Although not statistically significant, a general trend was observed between increased MDR1 mRNA expression [11] and Bliss independence synergy for the vincristine and lapatinib combination at standard library concentrations. (G) siRNA knockdown of MDR1 was confirmed by Western blotting to MDR1, as compared to GAPDH loading control, and by RT-PCR to *MDR1*, relative to *hTBP* control. Error bars represent s.d. of measurement replicates (*n* = 3). Also shown in the blot is basal MDR1 protein in WM451Lu cells, which is decreased compared to A375, correlating to decreased mRNA expression. (H) Western blotting confirmed over-expression of HA-tagged MDR1 in WM451Lu cells, relative to GFP control over-expression, and GAPDH loading control. (I) Synergistic interaction was seen between vincristine and 5μM lapatinib across a range of non-melanoma rapidly proliferating cells, including BxPc3 pancreatic cell line (*n* = 3), DU145 prostate cell line (*n* = 4), HeLa cervical cancer cell line (*n* = 3), MCF7 breast cancer cell line (*n* = 2), and PANC1 pancreatic cell line (*n* = 3). Much less synergy was seen in normal, more quiescent cells such as human fetal melanocytes (*n* = 4) and normal human fibroblasts (*n* = 2). Summary of maximum Bliss measurements for each cell line is shown on bottom right. Error bars represent s.d. of measurement replicates.
(TIF)

S4 Fig Summary of effects of drug combinations with PLX4720 across the melanoma cell line collection. (A) Hierarchical clustering of the Bliss synergy scores for all combinations with PLX4720, at both library concentrations (left), and the corresponding relative cell count values for each PLX4720-drug-cell line triad (right). (B) Top results of SAM analysis of combinations with PLX4720 specifically demonstrating synergy in the PLX4720-resistant cell lines.
(TIF)

S5 Fig Synergistic interaction between cediranib and PLX4720. (A) Isobologram demonstrating significant synergy between PLX4720 and cediranib, as shown in ISTMel1 cells. The Combination Index was 0.35, with a minimum of 0.01. (B) Synergy (Bliss ~30–40%) in ISTMel1 and RPMI7951 cells between cediranib and PLX4720 (top) and between cediranib and selumetinib (500nM, bottom). Error bars represent s.d. of measurement replicates (*n* = 2–3). (C) Cediranib induces a synergistic decrease in cell growth when in triple combination with PLX4720 and selumetinib (100nM). Error bars represent s.d. of measurement replicates (*n* = 2). (D) Cell cycle analysis by flow cytometry with propidium iodide. PLX4720 causes less G_1_ arrest in ISTMel1 cells compared to UACC62 cells. Addition of cediranib has no significant effect on cell cycle in either cell line. Data shown is average of three independent experiments. (E) No significant synergy was seen between PLX4720 and the canonical MDR inhibitor verapamil. Error bars represent s.d. of measurement replicates (*n* = 2).
(TIF)

S6 Fig Synergistic interaction between cediranib and PLX4720. (A) Summary table (top) of maximum Bliss synergy scores for interaction between the given RTK inhibitor and PLX4720 in ISTMel1 and RPMI7951 cells. Strongest synergies were observed with cediranib and tivozanib across the KDR/PDGFR class of targets, compared to more specific or broader spectrum or less potent inhibitors. Results for tivozanib (dual KDR/PDGFR inhibitor), crenolanib (PDGFRα/β inhibitor), and axitinib (KDR inhibitor) are shown below for ISTMel1 and RPMI7951 cell lines. Error bars represent s.d. of measurement replicates (*n* = 2). (B) Expression data for cediranib targets KDR and PDGFRα/β from the Center for Molecular Therapeutics(CMT)/Sanger database across melanoma cell lines screened in this study. Higher expression was seen in resistant lines ISTMel1 and RPMI7951. (C) Expression of KDR across the entire CMT/Sanger database of cell lines, showing melanoma lines have the highest expression of KDR compared to other tissues types.
(TIF)

S7 Fig Knockdown of cediranib targets in melanoma cells. (A) RT-PCR results of KDR expression normalized to *hTBP* in ISTMel1 cells following siRNA treatment to pooled KDR siRNA or two individual siRNAs. Knockdown efficiency correlated with synergy with PLX4720. Error bars represent s.d. of measurement replicates (*n* = 3). (B) RT-PCR results of PDGFRα, PDGFRβ, and KDR expression normalized to *hTBP* in ISTMel1 (left) and RPMI7951 (right) cells following siRNA treatment. Total siRNA concentration was normalized across single, double, and triple knockdown, using siRNA control reagent. Error bars represent s.d. of measurement replicates (*n* = 2).
(TIF)

S8 Fig Signaling pathway inhibition following cediranib and PLX4720 treatments. (A) No synergistic interaction was seen between MEK inhibition by selumetinib and PLX4720 in ISTMel1 (left) or RPMI7951 (right) cell lines. Error bars represent s.d. of measurement replicates (*n* = 2). (B) Western blotting to dpERK and pS6K following single or combined treatment with PLX4720 and cediranib in PLX4720 resistant ISTMel1 and PLX4720-sensitive UACC62. Despite intrinsic resistance to PLX4720, ISTMel1 showed near complete suppression of ERK activation after PLX4720, as in UACC62 cells. Combined cediranib and PLX4720 treatment caused further suppression of pS6K in ISTMel1 cells after 24 hours. (C) Akt pathway phospho-antibody array (Cell Signaling) results in resistant ISTMel1 and RPMI7951 cells and PLX4720 sensitive UACC62 cells, after 24 hours of treatment. ISTMel1 and RPMI7951 cells showed decreased pathway activation at multiple levels following combined treatment of cediranib and PLX4720. (D) Combined treatment of ISTMel1 and RPMI7951 cells with PLX4720 and various inhibitors of the mTor/Akt pathway showed only modest synergy (summary table below for maximum Bliss scores), suggesting the more pronounced synergy with cediranib is due to suppression of additional pathways. Error bars represent s.d. of measurement replicates (*n* = 2). (E) Western blotting to Akt pathway marker phospho S6K showed the pathway inhibitors had variable but expected effects on suppression of S6K activation but no correlation between pathway suppression and synergy with PLX4720.
(TIF)

S9 Fig Synergistic interaction between cediranib and PLX4720. (A) Interaction between PLX4720 and several Wnt pathway modulators including the β-catenin inhibitor FH535, GSK3β inhibitor TZDZ-8, and Wnt pathway inducer Wnti. (B) On-target pathway activity of PLX4720 and cediranib *in vivo*. Results are shown from lysates of ISTMel1 tumors after 72 hours of treatment. PLX4720-impregnated chow resulted in suppression of dpERK in tumors. Although Akt pathway suppression was seen in cell culture, significant suppression was not seen in tumors. Cediranib suppressed PDGFRα phosphorylation. Due to low expression levels, immunoprecipitation was performed to observe on-target suppression of pPDGFRβ and pKDR in tumors, detected by phospho-tyrosine antibodies following RTK immunoprecipitation. (C) Spider plots of all tumors from individual animals in the *in vivo* experiments using ISTMel1 or RPMI7951 cells for xenografts. (D) Animal weight during *in vivo* xenograft experiments showed no significant effects of single or combined cediranib and PLX4720 treatment. Error bars represent s.d. of measurement replicates (*n* = 7–9). (E) Synergistic interaction between PLX4720 and cediranib was observed in multiple non-melanoma *BRAF* ^*V600E*^-mutant cell lines, including NMC-G1 glioma (average Bliss synergy 31%), SW872 liposarcoma (average Bliss synergy 30%), and SW982 (average Bliss synergy 53%) sarcoma. No synergy was observed in the *BRAF* ^*V600E*^ colon cancer cell lines COLO201 and WiDr. Error bars represent s.d. of measurement replicates (*n* = 2–3).
(TIF)

S1 Table List of drugs used in study. Listing of 108 drugs used in building the combinatorial library, including current stage of clinical development, putative primary targets, and standard and low concentrations used.
(XLSX)

S2 Table Cell lines and key data. Listing of 36 cell lines used in the combination screen, including genotype of major oncogenes and key data from analysis of the vincristine-lapatinib and PLX4720-cediranib combinations discussed in detail in the manuscript.
(XLSX)

S3 Table Primary screening data. Full dataset of effects of drug combinations and individual drugs across all cell lines. Cell count data are presented as percent of DMSO control for single and combination drug effects. For combination data, values for Bliss synergy (positive values indicates synergistic cell killing) are also given. Cleaved PARP values are given as percent of DMSO control percent of nuclei positive for cleaved PARP; positive Bliss synergy indicates synergistic induction of cleaved PARP.
(XLSX)

S4 Table Melanoma patient sample data. Patients with metastatic melanoma containing BRAFV600E mutation (confirmed by genotyping) were enrolled on clinical trials for treatment with a BRAF inhibitor (BRAFi), or combined BRAF inhibition and MEK inhibition (BRAFi + MEKi). Patient, age, site of disease, treatment, maximal response, and duration of response are reported. SD = stable disease, PR = partial response, CR = complete response, sc = subcutaneous, n = nodal. lu = lung, li = liver, br = brain, b = bone.
(XLSX)
