# Correction: Landscape of Targeted Anti-Cancer Drug Synergies in Melanoma Identifies a Novel BRAF-VEGFR/PDGFR Combination Treatment

**DOI:** 10.1371/journal.pone.0306658

**Published:** 2024-07-01

**Authors:** Adam A. Friedman, Arnaud Amzallag, Iulian Pruteanu-Malinici, Subash Baniya, Zachary A. Cooper, Adriano Piris, Leeza Hargreaves, Vivien Igras, Dennie T. Frederick, Donald P. Lawrence, Daniel A. Haber, Keith T. Flaherty, Jennifer A. Wargo, Sridhar Ramaswamy, Cyril H. Benes, David E. Fisher

There is an error in S3 Table. Some data in the columns are duplicated. Please view the correct S3 Table below.

## Supporting information

S3 Table (XLSX)
