# Supplementary figures and images for: Landscape of Targeted Anti-Cancer Drug Synergies in Melanoma Identifies a Novel BRAF-VEGFR/PDGFR Combination Treatment
Source: PLoS One. 2015 Oct 13;10(10):e0140310. doi: 10.1371/journal.pone.0140310 (PMC4604168; doi:10.1371/journal.pone.0140310)

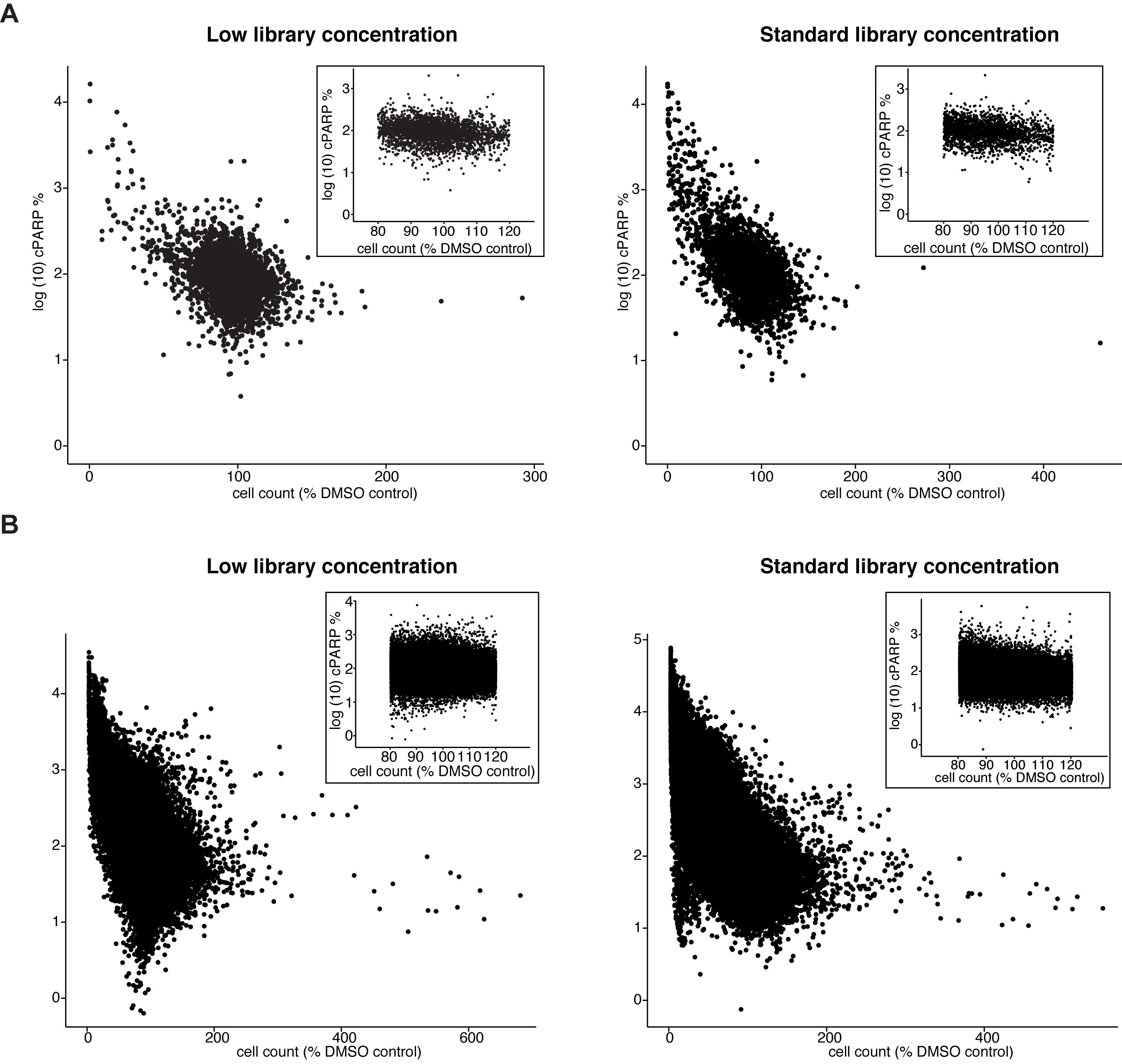

Supplement: S1 Fig — (A) Single drug results across all melanoma cell lines, plotted as the relationship between the normalized cell count (x axis) and the normalized and log-transformed percent of cPARP positive nuclei (y axis). Shown are the data for the low (left) and standard (right) library concentration. In general, single drugs that result in reduced viability also show an increase in cPARP percentage. Insets show population of single drugs resulting in relatively normal viability, some of which also show increase cPARP staining. (B) As in (A) but shown are results for all combinations across all melanoma cells lines. Also demonstrated in insets are specific population of drug-drug-cell line triads with relatively normal viability but increased cPARP staining. (TIF) [file pone.0140310.s001.tif]

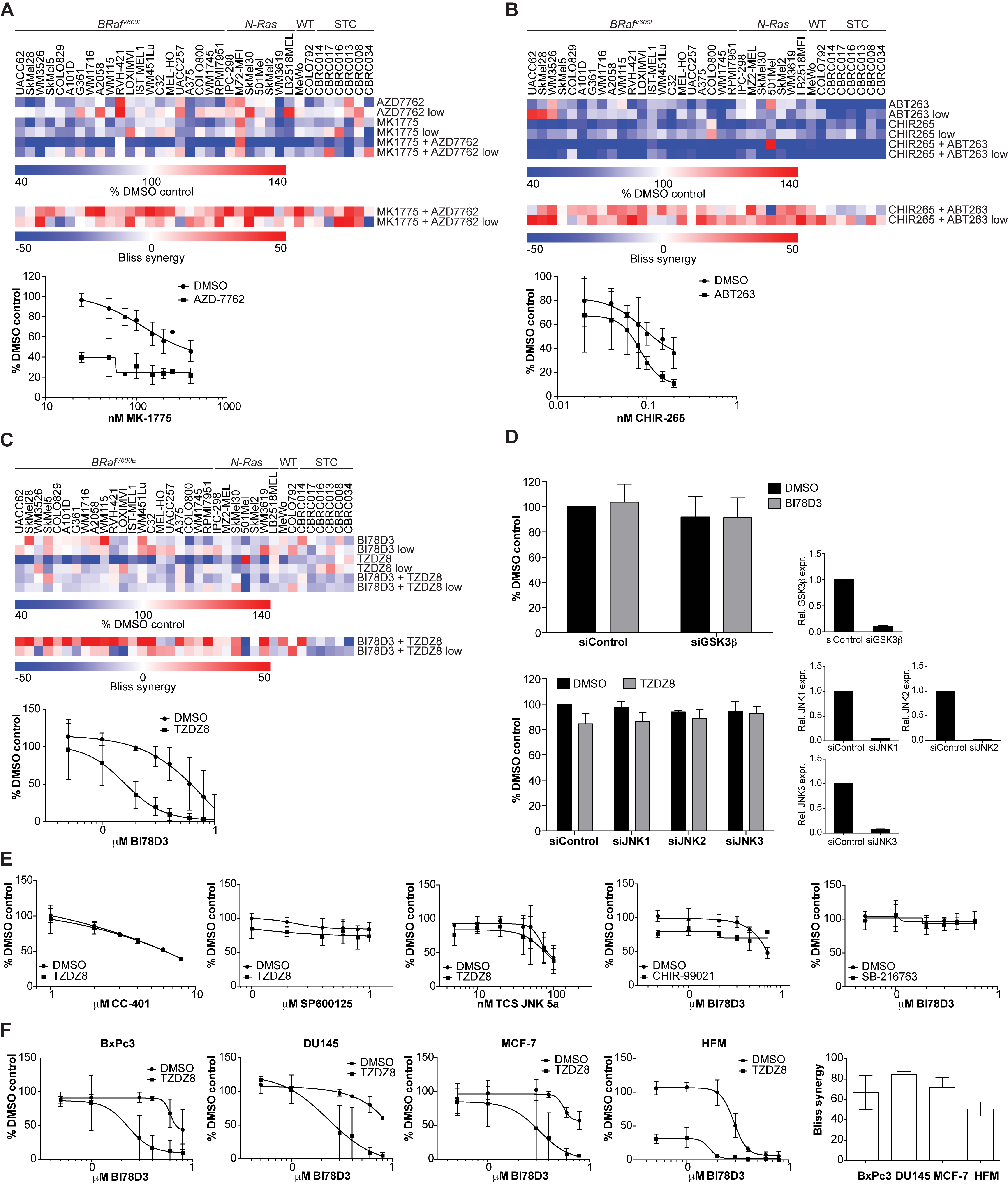

Supplement: S2 Fig — (A) AZD7762, a CHK1/2 inhibitor, displayed broad synergistic effects with MK1775, a WEE1 inhibitor, across multiple melanoma cell lines. This effect was confirmed in secondary experiments, below, showing synergistic interaction in A375 cells. Error bars represent s.d. of measurement replicates (n = 4). (B) CHIR265, an inhibitor of BRAF and other kinases, showed a synergistic interaction with ABT263, a BCL2 family inhibitor, at high doses of CHIR265; this effect was confirmed (below) in UACC62 cells. Error bars represent s.d. of measurement replicates (n = 3). (C) BI78D3, a JNK family inhibitor, showed strong synergy with TZDZ8, a GSK3β inhibitor, across multiple melanoma cell lines. This interaction was confirmed in secondary experiments (below) in A375 cells. Error bars represent s.d. of measurement replicates (n = 8). (D) siRNA knockdown of TZDZ8 target GSK3β (top, n = 5) or BI78D3 targets JNK1, JNK2, or JNK3 (bottom, n = 3) showed no synergy with 500nM BI78D3 or 5μM TZDZ8, respectively. Error bars represent s.d. of measurement replicates. RT-PCR confirming siRNA-mediated target knockdown is shown at right. Expression is normalized to ActB. Error bars represent s.d. of measurement replicates (n = 2). (E) No synergy was observed between 5μM TZDZ8 and a variety of other JNK inhibitors, including CC401 (n = 2), SP600125 (n = 3), and TCS JNK5a (n = 2). No synergy was observed between BI78D3 and other GSK3β inhibitors, including 1μM CHIR99021 (n = 4) and 1μM SB216763 (n = 3). Error bars represent s.d. of measurement replicates. (F) Synergistic interaction was seen between TZDZ8 and 5μM BI78D3 across a range of non-melanoma cells, including BxPc3 pancreatic cell line (n = 2), DU145 prostate cell line (n = 2), MCF7 breast cancer cell line (n = 2), and normal human fetal melanocytes (n = 2). Summary of maximum Bliss measurements for each cell line is shown on bottom right. Error bars represent s.d. of measurement replicates. (TIF) [file pone.0140310.s002.tif]

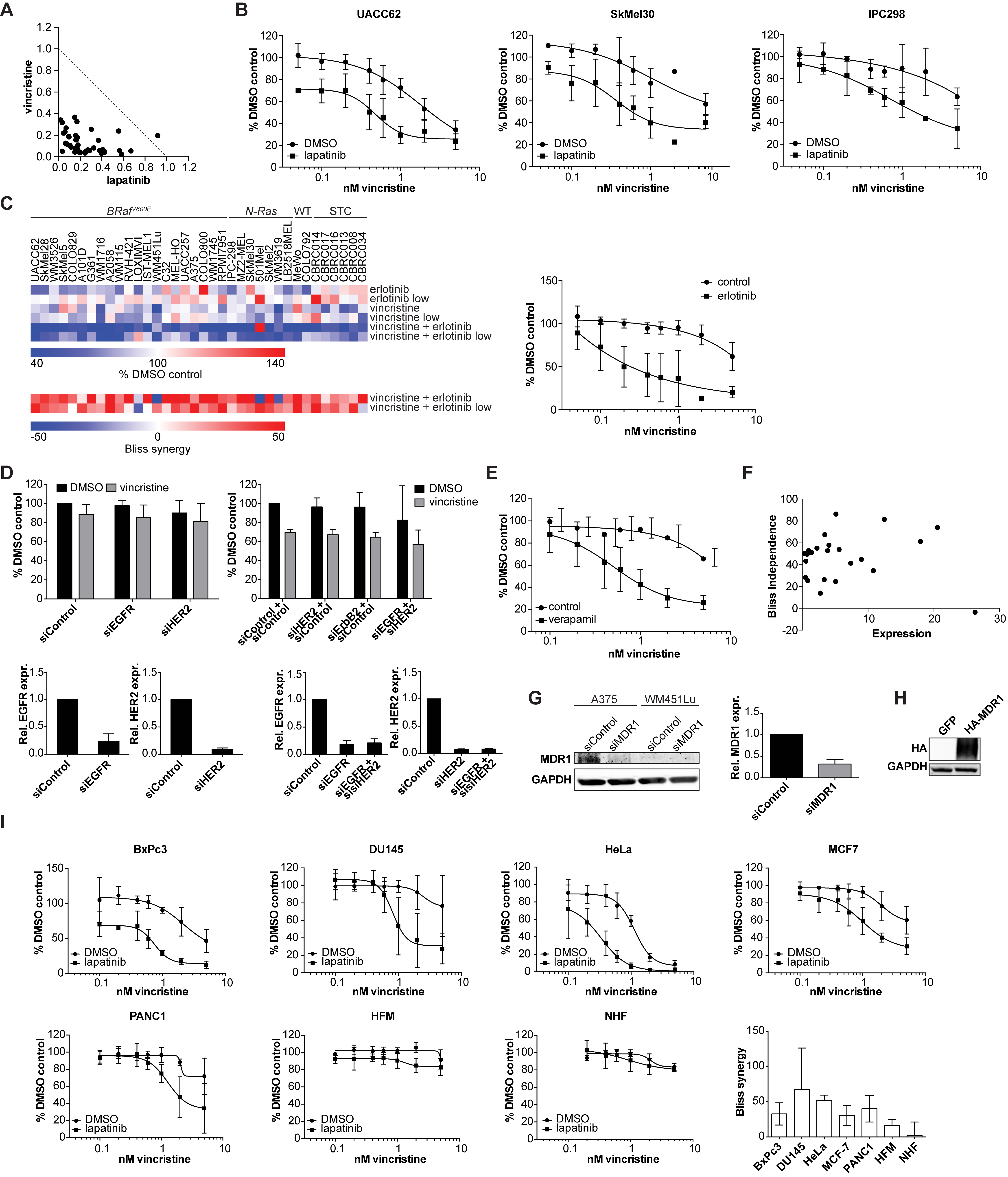

Supplement: S3 Fig — (A) Isobologram demonstrating significant synergy between vincristine and lapatinib, as shown in A375 cells. Combination Index was, on average 0.37, with minimum of 0.085. (B) Confirmation of synergy between vincristine and 5μM lapatinib in multiple other melanoma cell lines, including UACC62 (29% Bliss, n = 7), SkMel30 (36% Bliss, n = 3), and IPC298 (47% Bliss, n = 4). Error bars represent s.d. of measurement replicates. (C) Significant synergy was also seen in the primary screen across multiple melanoma cell lines between vincristine and erlotinib. This synergy was confirmed in secondary experiments in A375 cells (right). Error bars represent s.d. of measurement replicates (n = 3). (D) siRNA-mediated knockdown of lapatinib targets EGFR and HER2 demonstrated no synergy with 2nM vincristine either alone (left, n = 6) or in combination (right, n = 5). Error bars represent s.d. of measurement replicates. Target knockdown was confirmed by RT-PCR measurement and normalized to hTBP or ActB (below). Error bars represent s.d. of measurement replicates (n = 4). (E) Canonical MDR inhibitor verapamil (5μM) showed significant synergy with vincristine in A375 cells. Error bars represent s.d. of measurement replicates (n = 7). (F) Although not statistically significant, a general trend was observed between increased MDR1 mRNA expression [11] and Bliss independence synergy for the vincristine and lapatinib combination at standard library concentrations. (G) siRNA knockdown of MDR1 was confirmed by Western blotting to MDR1, as compared to GAPDH loading control, and by RT-PCR to MDR1, relative to hTBP control. Error bars represent s.d. of measurement replicates (n = 3). Also shown in the blot is basal MDR1 protein in WM451Lu cells, which is decreased compared to A375, correlating to decreased mRNA expression. (H) Western blotting confirmed over-expression of HA-tagged MDR1 in WM451Lu cells, relative to GFP control over-expression, and GAPDH loading control. (I) Synergistic interact [file pone.0140310.s003.tif]

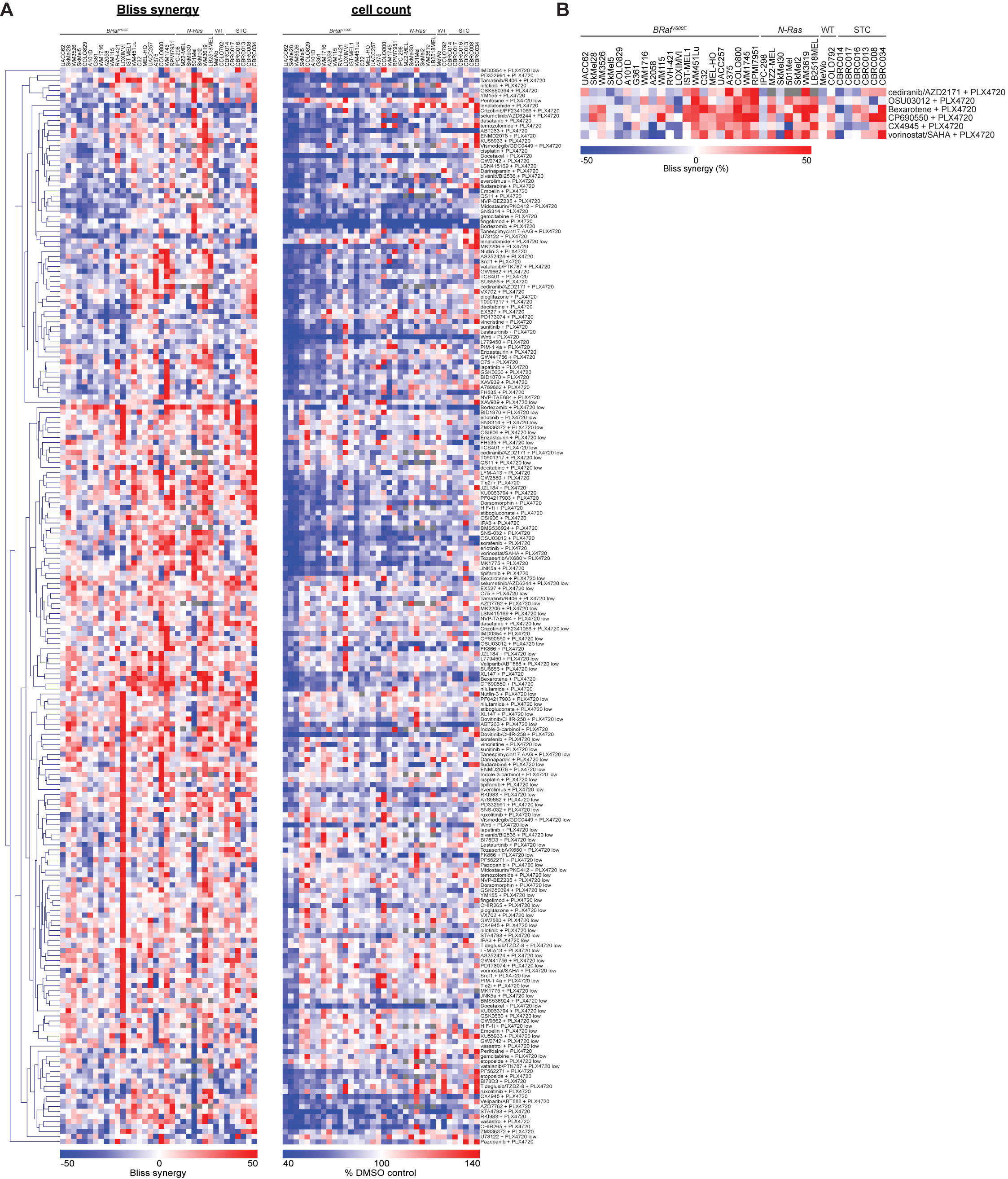

Supplement: S4 Fig — (A) Hierarchical clustering of the Bliss synergy scores for all combinations with PLX4720, at both library concentrations (left), and the corresponding relative cell count values for each PLX4720-drug-cell line triad (right). (B) Top results of SAM analysis of combinations with PLX4720 specifically demonstrating synergy in the PLX4720-resistant cell lines. (TIF) [file pone.0140310.s004.tif]

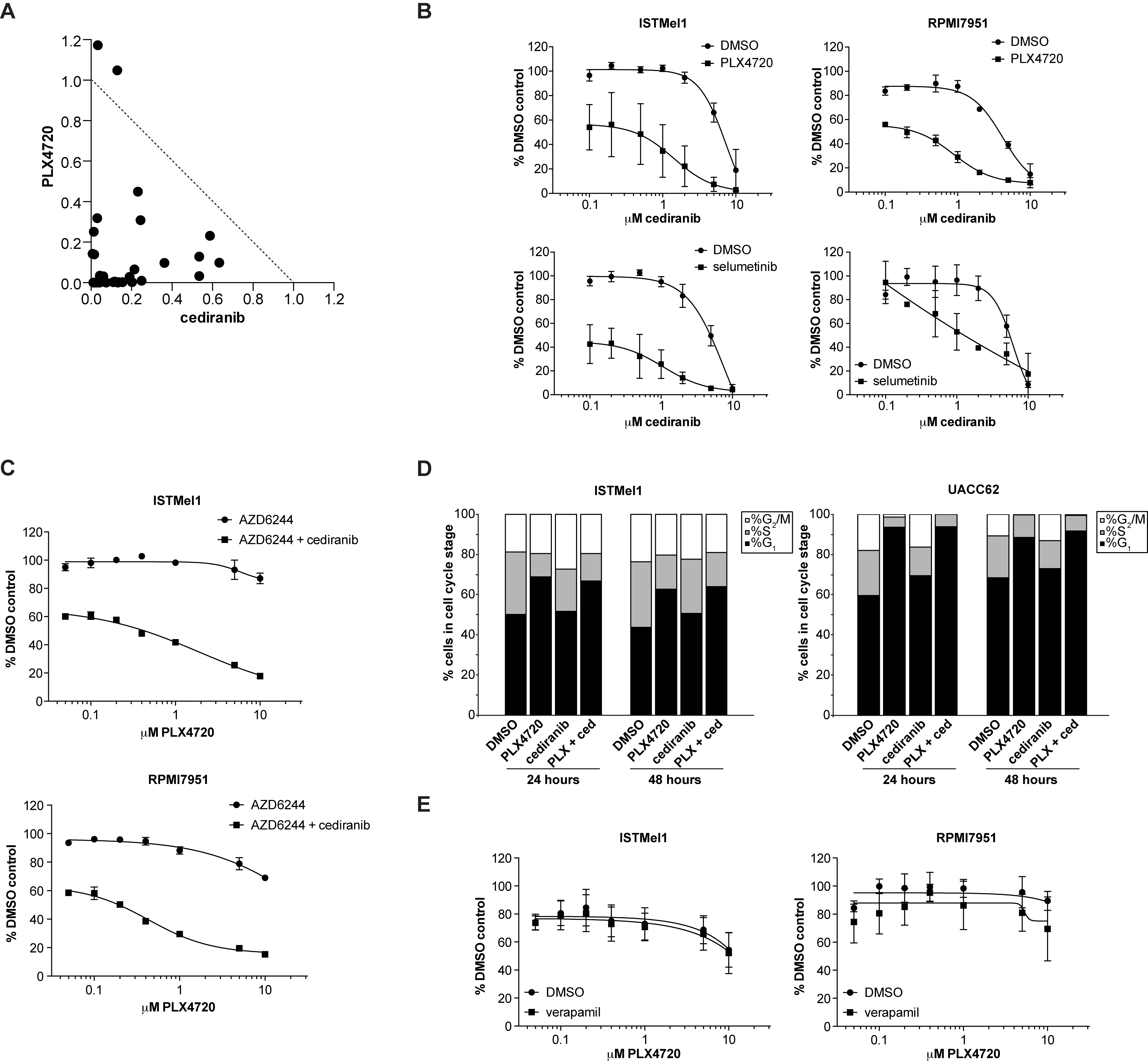

Supplement: S5 Fig — (A) Isobologram demonstrating significant synergy between PLX4720 and cediranib, as shown in ISTMel1 cells. The Combination Index was 0.35, with a minimum of 0.01. (B) Synergy (Bliss ~30–40%) in ISTMel1 and RPMI7951 cells between cediranib and PLX4720 (top) and between cediranib and selumetinib (500nM, bottom). Error bars represent s.d. of measurement replicates (n = 2–3). (C) Cediranib induces a synergistic decrease in cell growth when in triple combination with PLX4720 and selumetinib (100nM). Error bars represent s.d. of measurement replicates (n = 2). (D) Cell cycle analysis by flow cytometry with propidium iodide. PLX4720 causes less G1 arrest in ISTMel1 cells compared to UACC62 cells. Addition of cediranib has no significant effect on cell cycle in either cell line. Data shown is average of three independent experiments. (E) No significant synergy was seen between PLX4720 and the canonical MDR inhibitor verapamil. Error bars represent s.d. of measurement replicates (n = 2). (TIF) [file pone.0140310.s005.tif]

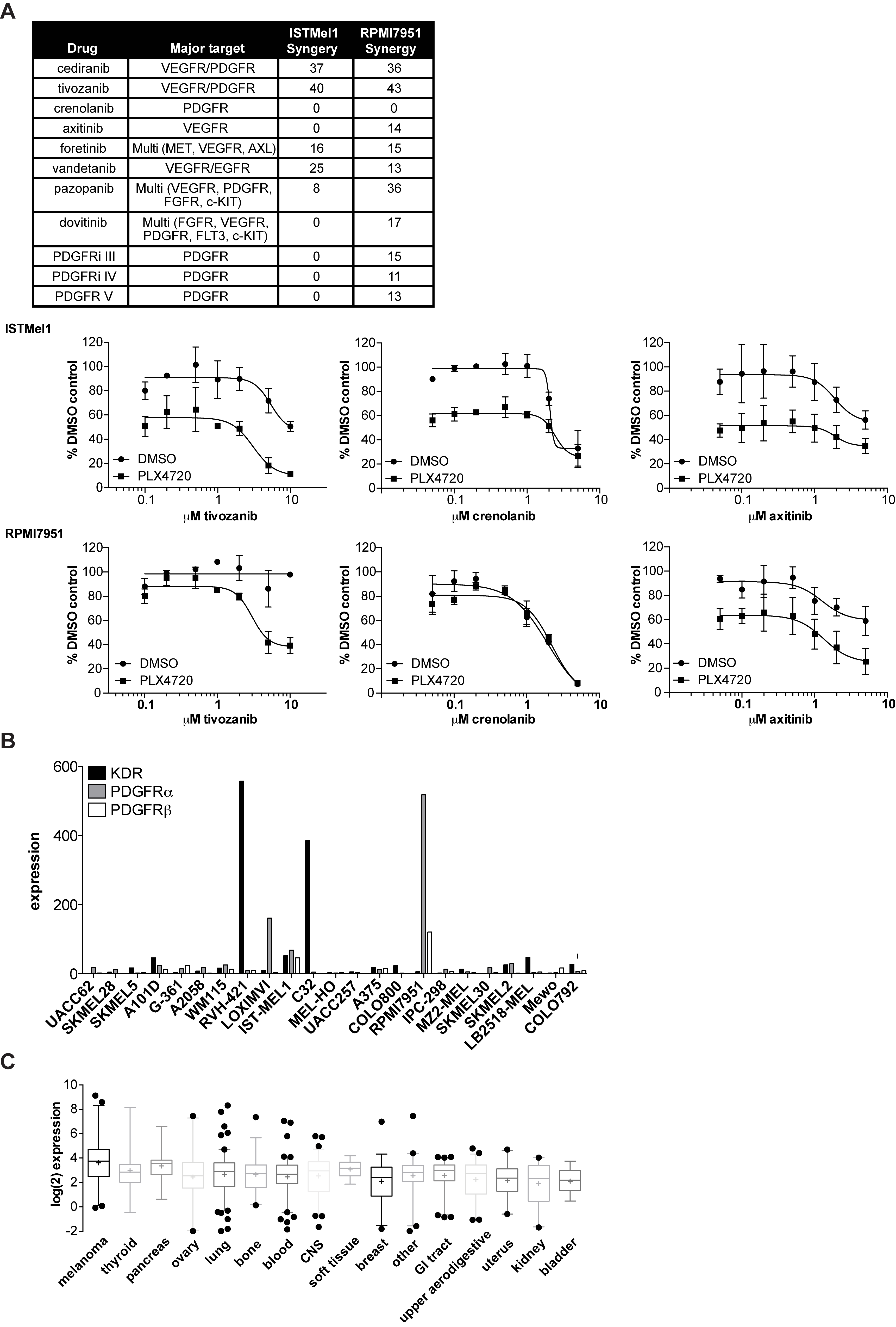

Supplement: S6 Fig — (A) Summary table (top) of maximum Bliss synergy scores for interaction between the given RTK inhibitor and PLX4720 in ISTMel1 and RPMI7951 cells. Strongest synergies were observed with cediranib and tivozanib across the KDR/PDGFR class of targets, compared to more specific or broader spectrum or less potent inhibitors. Results for tivozanib (dual KDR/PDGFR inhibitor), crenolanib (PDGFRα/β inhibitor), and axitinib (KDR inhibitor) are shown below for ISTMel1 and RPMI7951 cell lines. Error bars represent s.d. of measurement replicates (n = 2). (B) Expression data for cediranib targets KDR and PDGFRα/β from the Center for Molecular Therapeutics(CMT)/Sanger database across melanoma cell lines screened in this study. Higher expression was seen in resistant lines ISTMel1 and RPMI7951. (C) Expression of KDR across the entire CMT/Sanger database of cell lines, showing melanoma lines have the highest expression of KDR compared to other tissues types. (TIF) [file pone.0140310.s006.tif]

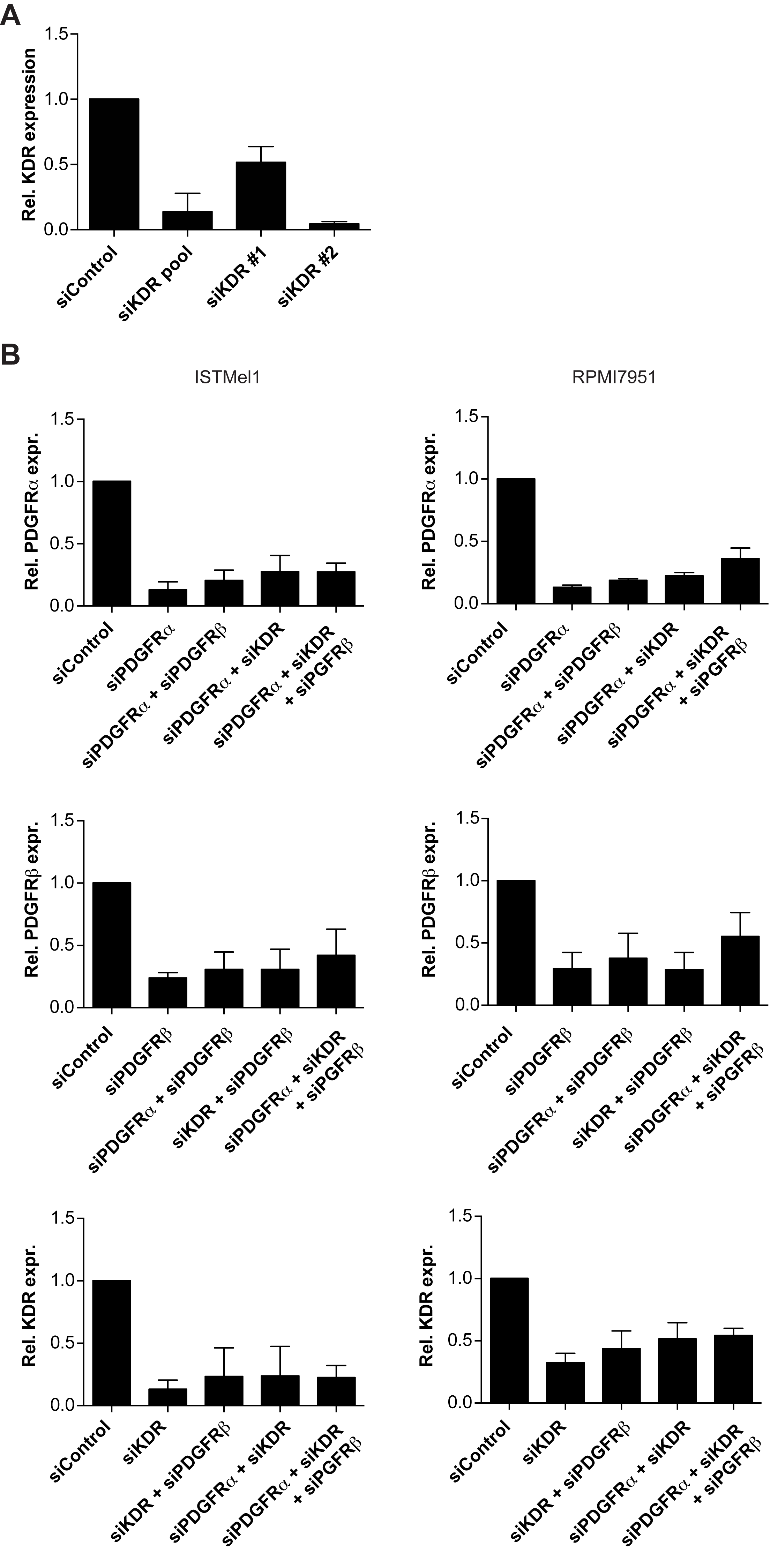

Supplement: S7 Fig — (A) RT-PCR results of KDR expression normalized to hTBP in ISTMel1 cells following siRNA treatment to pooled KDR siRNA or two individual siRNAs. Knockdown efficiency correlated with synergy with PLX4720. Error bars represent s.d. of measurement replicates (n = 3). (B) RT-PCR results of PDGFRα, PDGFRβ, and KDR expression normalized to hTBP in ISTMel1 (left) and RPMI7951 (right) cells following siRNA treatment. Total siRNA concentration was normalized across single, double, and triple knockdown, using siRNA control reagent. Error bars represent s.d. of measurement replicates (n = 2). (TIF) [file pone.0140310.s007.tif]

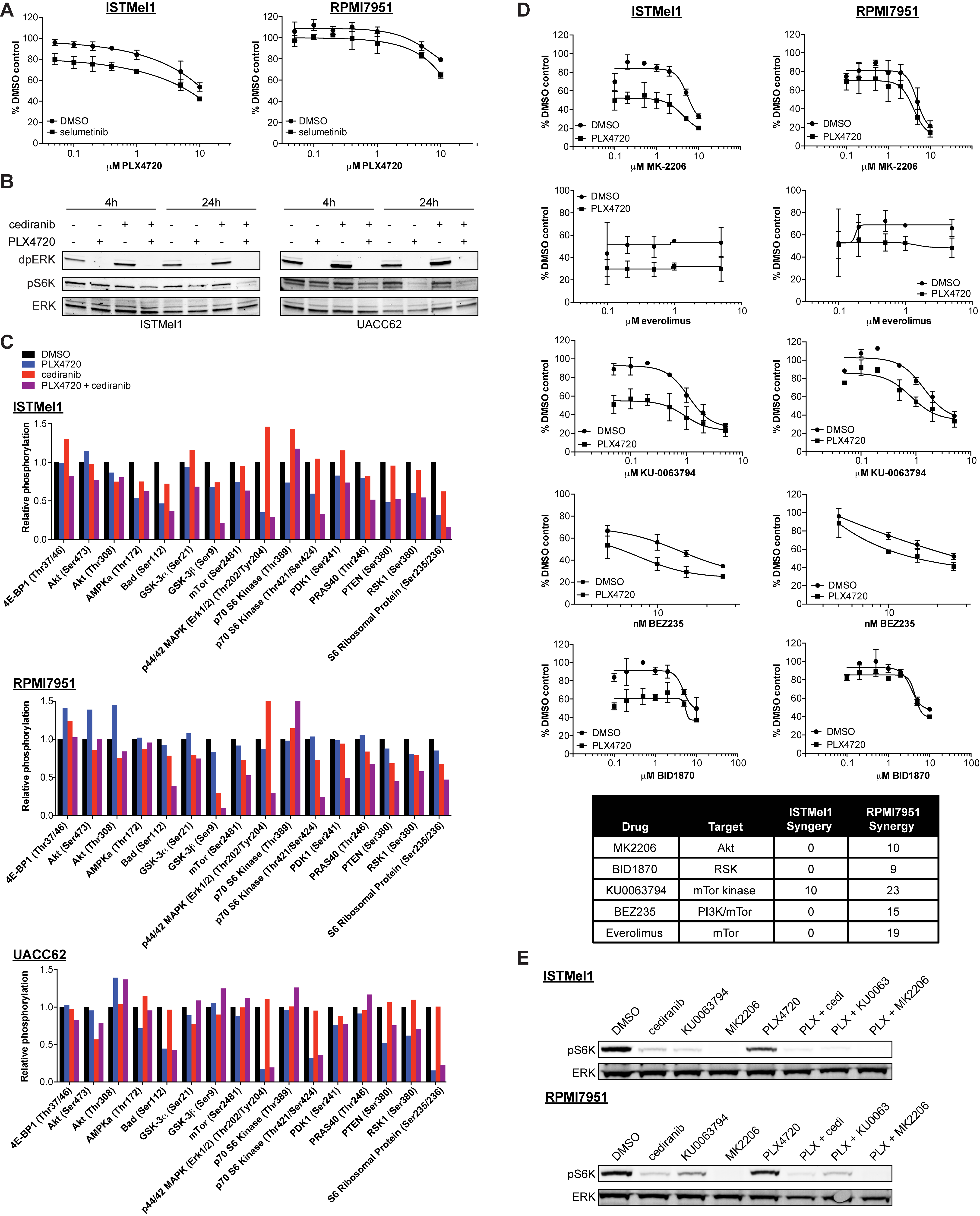

Supplement: S8 Fig — (A) No synergistic interaction was seen between MEK inhibition by selumetinib and PLX4720 in ISTMel1 (left) or RPMI7951 (right) cell lines. Error bars represent s.d. of measurement replicates (n = 2). (B) Western blotting to dpERK and pS6K following single or combined treatment with PLX4720 and cediranib in PLX4720 resistant ISTMel1 and PLX4720-sensitive UACC62. Despite intrinsic resistance to PLX4720, ISTMel1 showed near complete suppression of ERK activation after PLX4720, as in UACC62 cells. Combined cediranib and PLX4720 treatment caused further suppression of pS6K in ISTMel1 cells after 24 hours. (C) Akt pathway phospho-antibody array (Cell Signaling) results in resistant ISTMel1 and RPMI7951 cells and PLX4720 sensitive UACC62 cells, after 24 hours of treatment. ISTMel1 and RPMI7951 cells showed decreased pathway activation at multiple levels following combined treatment of cediranib and PLX4720. (D) Combined treatment of ISTMel1 and RPMI7951 cells with PLX4720 and various inhibitors of the mTor/Akt pathway showed only modest synergy (summary table below for maximum Bliss scores), suggesting the more pronounced synergy with cediranib is due to suppression of additional pathways. Error bars represent s.d. of measurement replicates (n = 2). (E) Western blotting to Akt pathway marker phospho S6K showed the pathway inhibitors had variable but expected effects on suppression of S6K activation but no correlation between pathway suppression and synergy with PLX4720. (TIF) [file pone.0140310.s008.tif]

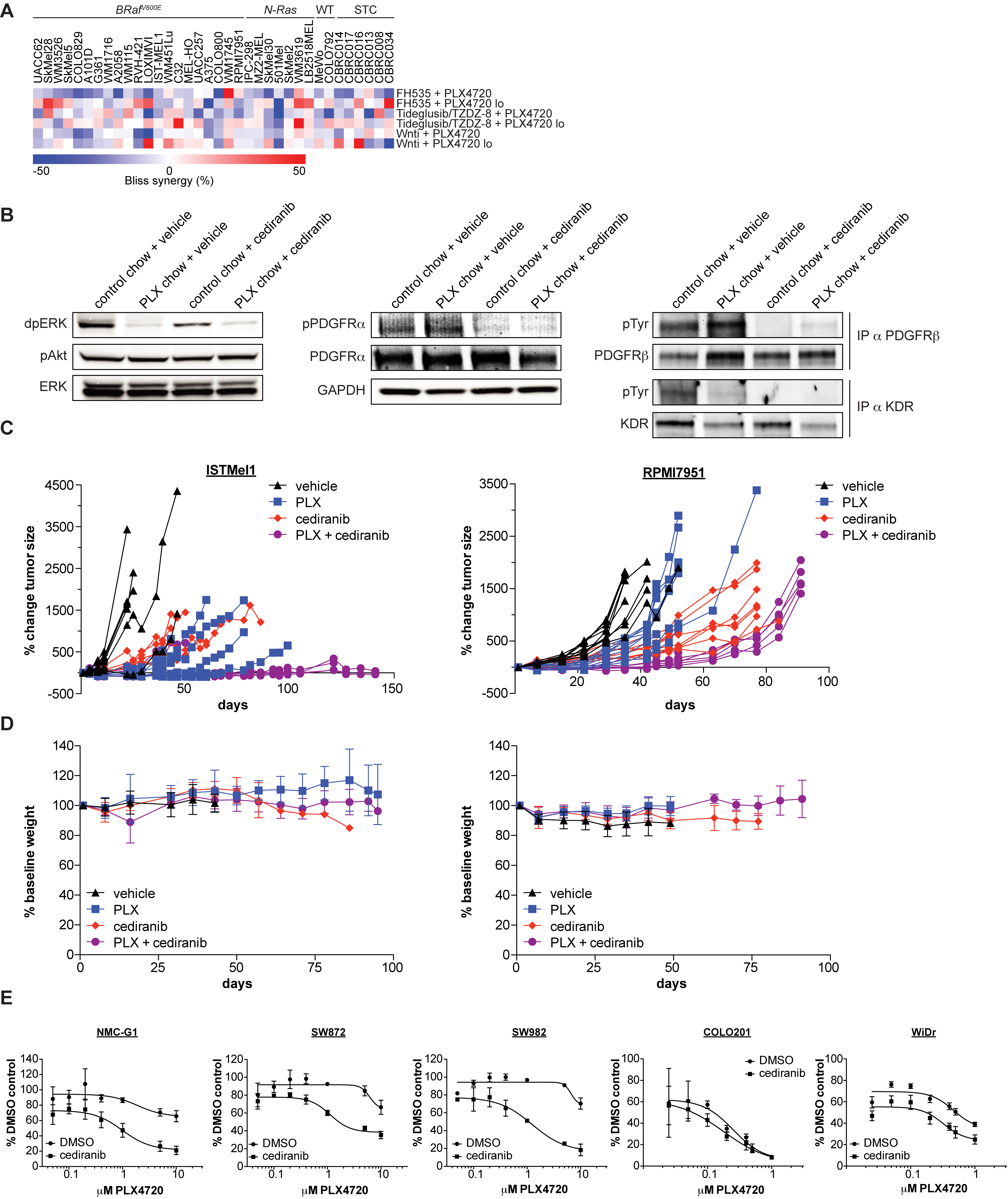

Supplement: S9 Fig — (A) Interaction between PLX4720 and several Wnt pathway modulators including the β-catenin inhibitor FH535, GSK3β inhibitor TZDZ-8, and Wnt pathway inducer Wnti. (B) On-target pathway activity of PLX4720 and cediranib in vivo. Results are shown from lysates of ISTMel1 tumors after 72 hours of treatment. PLX4720-impregnated chow resulted in suppression of dpERK in tumors. Although Akt pathway suppression was seen in cell culture, significant suppression was not seen in tumors. Cediranib suppressed PDGFRα phosphorylation. Due to low expression levels, immunoprecipitation was performed to observe on-target suppression of pPDGFRβ and pKDR in tumors, detected by phospho-tyrosine antibodies following RTK immunoprecipitation. (C) Spider plots of all tumors from individual animals in the in vivo experiments using ISTMel1 or RPMI7951 cells for xenografts. (D) Animal weight during in vivo xenograft experiments showed no significant effects of single or combined cediranib and PLX4720 treatment. Error bars represent s.d. of measurement replicates (n = 7–9). (E) Synergistic interaction between PLX4720 and cediranib was observed in multiple non-melanoma BRAF V600E-mutant cell lines, including NMC-G1 glioma (average Bliss synergy 31%), SW872 liposarcoma (average Bliss synergy 30%), and SW982 (average Bliss synergy 53%) sarcoma. No synergy was observed in the BRAF V600E colon cancer cell lines COLO201 and WiDr. Error bars represent s.d. of measurement replicates (n = 2–3). (TIF) [file pone.0140310.s009.tif]
